# Single‐Cell Transcriptomic Analysis of the Immune Response to COVID‐19 and Tuberculosis Coinfection

**DOI:** 10.1002/EXP.20240022

**Published:** 2025-05-08

**Authors:** Yi Wang, Maike Zheng, Yun Zhang, Yu Xue, Sibo Long, Chaohong Wang, Qing Sun, Jun Yan, Yiheng Shi, Bin Yang, Shang Ma, Tiantian Zhang, Lei Cao, Yan Chen, Wenfu Ju, Jing Zhang, Yan Zhao, Mengqiu Gao, Laurence Don Wai Luu, Xinting Yang, Guirong Wang

**Affiliations:** ^1^ Experimental Research Center Capital Center for Children's Health Capital Medical University Capital Institute of Pediatrics Beijing P. R. China; ^2^ Molecular Diagnostic Center Capital Center for Children's Health Capital Medical University Beijing P. R. China; ^3^ Department of Clinical Laboratory Beijing Chest Hospital Capital Medical University Beijing Tuberculosis and Thoracic Tumor Institute Beijing P. R. China; ^4^ Tuberculosis Department Beijing Chest Hospital Capital Medical University Beijing P. R. China; ^5^ Department of Emergency Beijing Chest Hospital Capital Medical University Beijing P. R. China; ^6^ School of Life Sciences University of Technology Sydney Sydney Australia; ^7^ School of Biotechnology and Biomolecular Sciences University of New South Wales Sydney Australia

**Keywords:** Coinfection, COVID‐19, Dysregulated immune response, scRNA‐seq, Tuberculosis

## Abstract

The immune characteristics and pathological mechanisms of COVID‐19 and tuberculosis coinfection are not well understood. Single‐cell RNA sequencing has emerged as a powerful tool for dissecting complex immune responses and cellular interactions in infectious diseases. Here, we employed scRNA‐seq, combined with laboratory examinations and clinical observations, to elucidate potential mechanisms of immunopathology and protective immunity in coinfected patients. Substantial alterations in immune cell populations in patients with severe coinfection were observed, characterized by severe lymphopenia and massive expansion of myeloid cells. Lymphocytopenia may have resulted from lymphocyte apoptosis and migration. Systemic upregulation of S100 family proteins, mainly released by classical monocytes, might contribute to inflammatory cytokine storm via *S100‐TLR4*‐*MyD88* signaling pathway in severely coinfected patients. Myeloid cells may contribute to immune paralysis in severe cases through expansion of myeloid‐derived suppressor cells and dysregulated dendritic cell function. The immune landscape of T cells in severe patients were featured by dysregulated Th1 response, widespread exhaustion and increased cytotoxic, apoptosis, migration and inflammatory states. We observed increased plasma cells and overexpression of B‐cell‐activation‐related pathways in severe patients. Together, we provide a comprehensive atlas illustrating the immune response to coinfected patients at the single‐cell resolution and highlight mechanisms of pathogenesis in severe patients.

## Graphical abstract

Understanding the immunological basis for severe outcomes in COVID‐19/Tuberculosis coinfection is critical. Our single‐cell analysis identifies key drivers: profound lymphocyte loss through combined apoptosis and altered trafficking, excessive S100 protein‐mediated inflammation contributing to cytokine storms, and systemic immune dysfunction characterized by exhausted T cells and suppressive myeloid populations. These findings pinpoint crucial nodes of immunopathology in severely coinfected individuals.

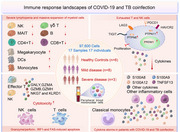

## 1 Introduction

Historically, *Mycobacterium tuberculosis* (*Mtb*), which causes tuberculosis (TB), was the leading cause of death from a communicable disease until the COVID‐19 pandemic. Previous studies found altered immune cell subsets in TB, including a depletion of natural killer (NK) cells in peripheral blood [1] and an enrichment of granzyme K‐expressing CD8^+^ T cells in pleural fluids [2]. Severe TB is characterized by lymphopenia, immune exhaustion, immune paralysis, and inflammatory cytokine storms, resulting in immune system damage [1]. These may lead to coinfection of bacteria or viruses in TB cases. Coinfection of SARS‐CoV‐2 and *Mtb* has been reported in some countries [3], leading to an increased risk of severe disease and death. A recent meta‐analysis showed that the fatality rate for active TB‐COVID‐19 coinfection was 10.6% (95% CI, 7.9–13.6%), significantly higher than the 0.68% for COVID‐19 alone [4]. The study also indicated that TB‐COVID‐19 coinfection increases hospitalization risks, prolongs recovery periods, and accelerates mortality compared to COVID‐19 alone [4].

In 2020, COVID‐19 replaced TB as the leading cause of death from an infectious disease. Characteristic features of COVID‐19 include elevated serum proinflammatory cytokines, lymphopenia, impaired B cell activation and altered lymphocyte function [5]. Advanced age, obesity, high blood pressure, and chronic lung disease have been associated with poorer outcomes in COVID‐19 [6]. Approximately 14.3% of severe COVID‐19 patients had secondary bacterial infections [7], with TB identified as a risk factor for increased disease severity [8].

TB and COVID‐19 share transmission characteristics and exhibit dysregulated immune responses [1, 9, 10]. While the immune response in diseases has been well characterized, data on the impacts of COVID‐19 on TB outcomes and vice versa are limited. Petrone et al. [11] demonstrated that patients with COVID‐19 and TB coinfection had a decreased capacity to establish an immune response to COVID‐19 while Riou et al [12]. observed that differentiated CD4^+^ T cells in coinfected patients had a reduced ability to respond to both COVID‐19 and *Mtb*. Najafi‐Fard et al. [13] also found decreased SARS‐CoV‐2 and *Mtb*‐specific immune responses in coinfected patients. Although these studies provide important insights, a comprehensive immune landscape for COVID‐19 and TB coinfection is critical for developing better treatments and reducing the risk of hospitalization or fatality in coinfected patients.

Single‐cell RNA sequencing has emerged as a powerful technology for revealing complex immune responses and cellular interactions in infectious diseases [1, 14–16]. To elucidate the complex host response to SARS‐CoV‐2 and *Mtb* coinfection, we performed single‐cell transcriptomics of peripheral blood mononuclear cells (PBMCs) from coinfected patients. Our results provide a comprehensive, unbiased survey of the immunological response to COVID‐19 and TB coinfection, enhancing our understanding of the pathogenic host immune response.

## 2 Results

### 2.1 Single‐Cell Transcriptomic Analysis of COVID‐19‐TB Coinfected Patients

**FIGURE 1:**
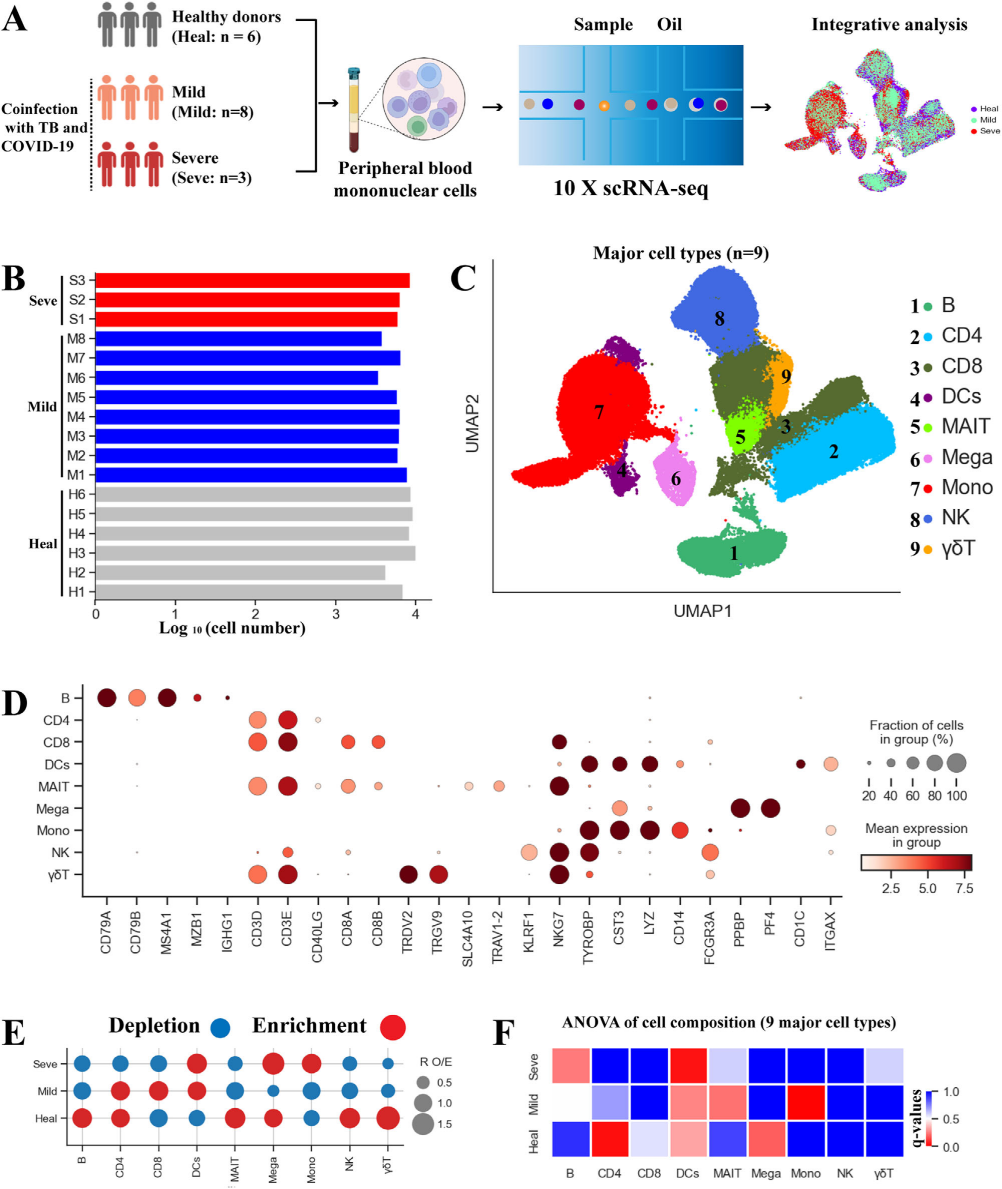
Overall results and study design of single‐cell PBMC transcriptomic profiling for TB and COVID‐19 coinfected cases. (A) Schematic of the study design. 17 samples were collected from 17 individuals, including 6 healthy donors (Heal) and 11 cases with COVID‐19 and TB coinfection (8 with mild symptoms (Mild) and 3 with severe symptoms (Seve)). (B) Bar plot illustrating the number of cells from each sample. (C) UMAP clustering of the 9 major cell types identified from 17 samples. Each dot represents a single cell that is colored based on cell type. (D) Dot plots of selected marker genes (Rows) for 9 major cell types (Columns). (E) Disease preference for each major cell type as calculated with *R*_O/E_. (F) Heatmap showing the ANOVA q values from the analysis of differences in cell composition between disease types.

To explore the immunological mechanism and pathogenesis of COVID‐19 and TB coinfection, we generated a scRNA‐seq dataset from 17 PBMC samples including 6 healthy controls (Heal) [1] and COVID‐19‐TB coinfected patients with varying clinical severity (8 mild and 3 severe cases) (Figure 1A). Laboratory and clinical findings for enrolled patients are provided in Table S1, Supporting Information. Using the 10× Genomics scRNA‐seq platform, 113,601 cells were obtained from 17 samples. After stringent quality control (See methods), 97,600 single cells remained, with an average of 6158 UMIs (unique molecular identifiers), representing 1987 genes (Figure 1B, Figure S1A, Supporting Information). We identified nine major cell types, including B, CD4^+^ T, CD8^+^ T, γδT, MAIT (mucosal‐associated invariant T cells), NK (natural killer cells), DCs (dendritic cells), monocytes (Mono), megakaryocyte (Mega), which covered the major cell clusters in peripheral blood (Figure 1C,D, Table S2, Supporting Information). Most cell clusters contained cells from different coinfected patients, suggesting common immunological features in these patients (Figure S1B–G, Supporting Information).

We found notable differences based on the UMAP (uniform manifold approximation and projection) projection (Figure 1A). The severity preference for each cell cluster was calculated using *R*_O/E_, which compares the observed cell count to the randomly expected cell count, removing the technical variations on tissue preference estimation (Figure 1D) [1]. In severe coinfected patients, we observed a depletion of lymphocyte cells, including B (except plasma cells), CD4^+^ T, CD8^+^ T, γδ T, MAIT, and NK cells (Figure 1E), indicating that lymphopenia is a prominent characteristic in these patients. Conversely, all clusters of myeloid cells were enriched in severe patients, including Mono, Mega, and DCs (Figure 1E, Figure S1G, Supporting Information). Interestingly, the lymphopenia phenomenon and the elevated myeloid cells were also found in severe TB or COVID‐19 patients [1] but not in coinfected patients with mild disease (Figure 1E, Figure S1G, Supporting Information). This suggests a distinct immune features between mild and severe cases. We further investigated the association between cell compositional changes with disease severity using ANOVA, and found that B and monocyte clusters were notably associated with severe and mild disease, respectively (Figure 1F). Collectively, these findings highlight key immunological changes in coinfected patients, particularly the lymphopenia in severe coinfection.

### 2.2 Association of Disease Severity With PBMC Compositions

**FIGURE 2:**
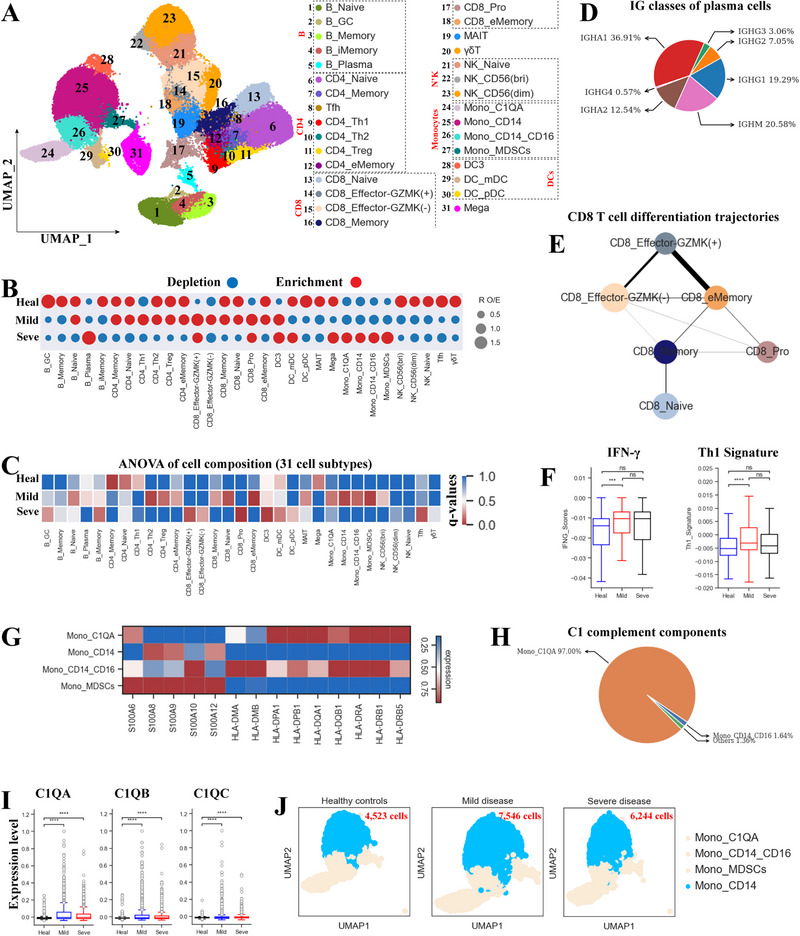
Associations between disease severity and PBMC composition. (A) UMAP clustering of the 31 cell subtypes (right row) identified from 17 samples. Each dot represents a single cell that is colored based on cell type. (B) Disease preference for each cell subtype as calculated with *R*_O/E_. C) Heatmap showing the ANOVA q‐values from the analysis of differences in cell subtype composition between disease types. (D) Pie chart showing the plasma cell heavy chain classes from Seve group. (E) CD8^+^ T cell pseudo‐time PAGA analysis: the associated cell type and the corresponding status are listed. (F) Box plots showing the IFNG and Th1 signature gene expression in Th1 cells across conditions. (G) Heatmap showing expression of selected marker genes for 4 monocyte subtypes. (H) Pie charts illustrating the relative proportion that each cell type contributes to the C1 complement components. (I) Box plots showing C1QA, C1QB, C1QC expression in Mono_ C1QA across groups. (J) UMAP projection of peripheral Mono_CD14 cells from each group.

The 9 major cell types were further subdivided into 31 different cell subtypes (Figure 2A, Table S2, Supporting Information), including 5 B cell subtypes (Figure S2A,B, Supporting Information), 7 CD4^+^ T cell subtypes (Figure S2C,D, Supporting Information), 6 CD8^+^ T cell subtypes (Figure S2E,F, Supporting Information), 3 NK cell subtypes (Figure S2G,H, Supporting Information) and 8 myeloid cell subtypes (Figure 2I,J) [17]. We then investigated the compositional change in these subsets (Figure 2B, Figure S3, Supporting Information) and their association with disease severity (Figure 2C). *R*_O/E_ analysis revealed that most B cell subtypes were depleted in the severe coinfected group, with only plasma B cells (B_Plasma) being enriched (Figure 2B). B_Plasma cells, highly expressing *CD38*, *MZB1*, *XPB1*, *PRDM1*, and *JCHAIN* (Figure S2B, Table S2, Supporting Information), were significantly associated with the mild coinfected group (Figure 2C). The increased plasma cells in coinfected patients (Figure S3F, Supporting Information), especially for those with severe disease, might induce neutralizing antibodies against SARS‐CoV‐2 (Figure S3F, Supporting Information). Genes encoding immunoglobulin constant regions (*IgM*, *IgG1*, *IgG2*, *IgA1*, and *IgA2)* were highly expressed in B_Plasma subtype, consistent with its function antibody production (Figure 2D). Interestingly, Ig gene expression was higher in severe coinfected patients than those with mild disease and healthy donors (Figure S4A, Supporting Information), implying that severe cases may have high levels of SARS‐CoV‐2 antibodies, consistent with previous reports [15].

Pseudo‐time analysis indicated that increased plasma B cells were likely derived from memory B cells (B_Memory) (Figure S4B, Supporting Information), which was the unique source for plasma B cells (Figure S4B, Supporting Information). Transition from B_Memory to B_Plasma was confirmed using partition‐based graph abstraction (PAGA) map (Figure S4B, Supporting Information). We observed that memory B clusters were decreased in coinfected patients and were associated with severe disease (Figure 2B,C, Figure S3F, Supporting Information). The increase in plasma B cells and decrease in precursor B_Memory cells indicate stronger B cell responses in the severe coinfected group. These results reveal an association between B cell subtypes with COVID‐19‐TB coinfection‐associated disease.

T cell sub‐clusters also displayed distinct associations with disease severity in coinfected patients (Figure 2C), indicating a complex T cell response induced by *Mtb* and SARS‐CoV‐2 coinfection. Most T cell subtypes were decreased in the severe coinfected group but increased in coinfected patients with mild disease (Figure 2B). However, two CD8^+^ T subtypes (CD8_Pro and CD8_Effector‐GZMK^(+)^) were elevated in all coinfected patients (Figure 2B, Figure S3F, Supporting Information). CD8_Pro cell subtype, characterized by proliferative markers (e.g. *MKI67* and *TYMS*) (Figure S2 and Table S2, Supporting Information), showed a notable association with severe coinfection (Figure 2C). PAGA analysis indicated that CD8_Pro were likely derived from memory CD8^+^ T cells (CD8_Memory) (Figure 2E), and serves as an intermediate state connecting to most CD8^+^ T subtypes from naïve to activated CD8^+^ T cell subsets. CD8_Memory cells were significantly depleted in severe coinfected patients compared to controls (Figure 2B, Figure S3F, Supporting Information). The increase in proliferative CD8^+^ T cells (CD8_Pro) and decrease in precursor CD8_Memory cells suggest stronger CD8^+^ T cell responses in severe disease, potentially causing pathogenic injury.

Th1 cells (CD4_Th1), marked by *IFNG*, *TBX21*, *MKI67*, and *ISG20* (Figure S2, Table S2, Supporting Information), are important in controlling *Mtb* infection through cytokine release (e.g. IFN‐γ) [1]. Mild coinfected patients showed an obvious increase in Th1 cells, whereas their abundance decreased in severe disease (Figure 2B). Further analysis revealed that Th1 cells were derived from CD4^+^ T memory (CD4_Memory) and effector memory (CD4_eMemory) cells, with CD4_eMemory being the primary source (Figure S4C, Supporting Information). CD4_eMemory cells were elevated in the mild coinfected group but decreased in the severe group. The depletion of Th1 cells and their precursor cells (CD4_eMemory) indicates a dysregulated Th1 response in severe coinfected patients. IFN‐γ expression was significantly increased in the mild coinfected group compared to controls. There was no increase in IFN‐γ expression or other Th1 signatures in the severe coinfected group (Figure 2F, Figure S4D,E, Supporting Information). This suggests that the decreased IFN‐γ and Th1 signatures may contribute to an ineffective immune response to TB in severe coinfected patients.

Unlike most CD4^+^T and CD8^+^T subtypes that were elevated in the mild coinfected group and depleted in the severe group, all NK subsets, γδT and MAIT cells were decreased in coinfected patients (Figure 2B) but differed in their association with disease severity (Figure 2C). The depletion of NK, γδT and MAIT cells in COVID‐19 or TB infection, particularly in severe patients, has been previously reported [1, 15], suggesting a similar response in single TB or COVID‐19 infection and coinfection.

Contrary to decreased lymphocytes in severe coinfected patients, most myeloid cell subtypes were increased (Figure 2B). Similar findings have been observed in single TB and COVID‐19 patients with severe disease [1, 15]. Further analysis found that myeloid cell subtypes displayed different associations with coinfected patients (Figure 2C), i.e. DC subtypes had notable association with mild disease while monocytes were associated with severe disease. In particular, a specific monocyte subtype known as MDSCs (myeloid‐derived suppressor cells) (Mono_MDSCs) was identified (Figure 2A). This subtype is characterized by upregulation of calprotectin (e.g. *S100A6*, *S100A9*, *S100A8*) and downregulation of MHC‐II molecules (especially for *HLA‐DR* molecules) (Figure 2G) [18]. PAGA analysis revealed that MDSCs seemed to be derived from classical monocytes (Mono_CD14) (Figure S4F, Supporting Information), in line with previous results that monocytic MDSCs in PBMCs have the CD14^+^ phenotype [19]. MDSCs, increased in various inflammatory conditions and capable of suppressing T cell responses [18, 20], were obviously enriched in the severe coinfected group (Figure 2B, Figure S3F, Supporting Information). These results suggest that monocytes in the severe coinfected group highly resembled MDSCs. Besides MDSCs, we identified another specific monocyte subtype (Mono_C1QA), marked by high expression of *C1QA/B/C* (Figure 2A, Figure S2, Supporting Information). Further analysis confirmed that this cluster was the major peripheral source of C1 complement (Figure 2H, Figure S4G, Supporting Information). These genes, encoding C1 complement components (*C1QA*, *C1QB*, and *C1QC*), were significantly elevated in the coinfected group versus controls, suggesting that C1 complement components may have diagnostic value for COVID‐19 and TB coinfection. Finally, differential UMAP projection patterns for classical monocytes (Mono_CD14) between healthy controls and coinfected patients indicated altered transcriptomic characteristics (Figure 2J).

### 2.3 The *S100* family Proteins, Expressed Mainly by Classical Monocytes, Contribute to Cytokine Storms in Severe Coinfection

**FIGURE 3:**
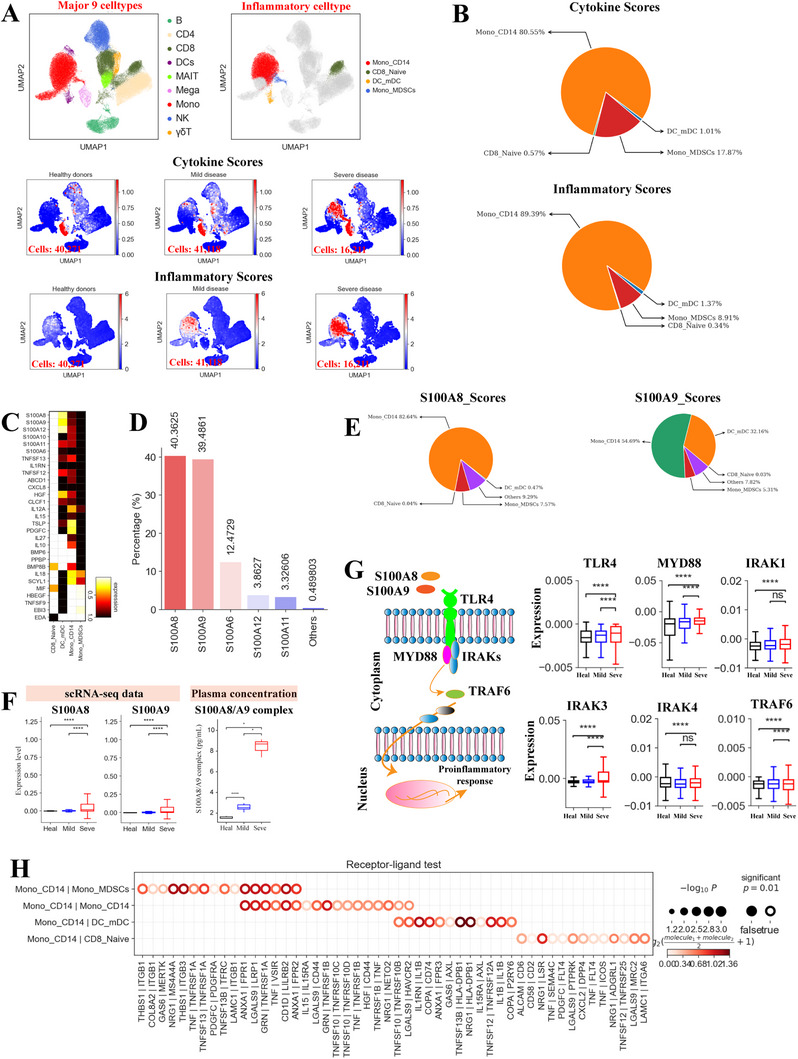
Contribution of *S100* family proteins to cytokine storms in severe infection. (A) UMAP plots: The colour for each panel shows the major cell types (Top left panel), inflammatory cell types (Top right panel), cytokine scores (Middle panel) and inflammatory scores (Bottom panel). (B) Pie charts illustrating the relative proportion that each inflammatory cell type contributes to the cytokine score (Top panel) and inflammatory score (Bottom panel). (C) Heatmap showing expression of cytokines in each of the hyperinflammatory cell subtype. (D) Bar charts illustrating the relative contribution from the top 5 cytokines in the severe coinfected group. (E) Pie charts illustrating the relative percentage contribution of each cell type to the *S100A8*‐ and *S100A9*‐score. (F) Box plots depicting the expression of *S100A8*/*A9* across each group (Left panel), box plots showing plasma profiling of *S100A8/A9* complex across different groups (Right panel). (G) The expression analysis of *S100A8*/*A9*‐*TLR4*‐*MyD88* pathway. (H) Dot plot depicting Mono_CD14 and interactions with selected inflammatory cell types in coinfected patients with severe disease. The size of the circle indicates *p* values with the scale shown on the right.

We next investigated the possible sources of cytokine release using pre‐defined cytokine and inflammatory genes [1] (Table S3, Supporting Information) to calculate cytokine and inflammatory score for each disease group and cell type (Figure S5, Supporting Information). These interrelated scores were used to assess the potential contribution to inflammatory response for each cell type. We observed significantly upregulated expression of cytokine and inflammatory genes in coinfected patients, especially in those with severe disease (Figure 3A, Figure S5A, Supporting Information), implying a potential inflammatory cytokine storm in patients with severe disease. Four subtypes, including two from monocytes (Mono_CD14 and Mono_MDSCs), one from CD8^+^ T cells (CD8_Naïve) and one from DC cells (DC_mDC), had significantly higher inflammatory and cytokine scores in the coinfected severe group (Figure S5B–G, Supporting Information), suggesting these cells are the primary contributors to the inflammatory cytokine storm. Further analysis revealed that classical monocytes (Mono_CD14) were the major source of inflammatory storm in the severe coinfected group (Figure 3B). Interestingly, CD14‐expressing monocytes (Mono_CD14) have been identified as key contributors of cytokine storm in severe TB or COVID‐19 patients [1].

The percentage of these four identified cell subsets were significantly increased in the severe coinfected group (Figure S6A, Supporting Information). These inflammatory cell subtypes showed different enrichment patterns in coinfected patients (Figure 2B). For each inflammatory cell subtype in severe coinfected patients, we investigated their inflammatory gene signature and identified unique cytokine expression patterns (Figure 3C), such as *S100A6*, *S100A8*, *S100A9*, *TNFSF13*, *CXCL8*, *IL10*, *PPBP*, *MIF* etc. In addition, we observed high expression of characteristic pro‐inflammatory cytokines (e.g. *TNF*, *CXCL1*/2/3, *CCL8*, *IL1A*, *PF4* etc.) in severe patients (Figure S6B, Supporting Information). These data suggest that various mechanisms potentially contributed to cytokine storm in the severe group. Further analysis found that >99% of cytokine scores in severe patients were due to the top 5 most highly expressed cytokines (*S100A6*/*8/9/11/12*) (Figure 3D), mainly secreted by classical monocytes (Mono_CD14) (Figure 3E, Figure S6C, Supporting Information). This suggests a central role for *S100A6/8/9/11/12* cytokines and Monocyte_CD14 subtype in driving inflammatory storm. Interestingly, significantly upregulated expression of *S100A6*/*8/9/11/12* genes were observed in the severe coinfected group (Figure 3F, Figure S6D, Supporting Information), further validating our findings. For this cohort, we measured cytokine levels in plasma, which confirmed that severe patients had higher levels of the S100A8/A9 complex (Figure 3F). In addition, we found that classical monocytes (Mono_CD14) highly expressed various cell‐type‐specific cytokines (e.g. *S100A6*/*8/9/10/11/12*, *CXCL8*, *CLCF1* and *IL1RN* etc.) (Figure 3C), further confirming their important role in driving the inflammatory storm. These results suggest the importance of classical monocytes for devising potential therapeutic methods to alleviate immunopathogenesis in coinfected patients with severe disease.

Among the top 5 cytokines, *S100A8/9* may play a key part in triggering the cytokine storm in the severe coinfected group as they contributed to ∼80% of the cytokine score (Figure 3D). *S100A8/9*, which are highly expressed during inflammation, modulate the inflammatory response by inducing cytokine release and stimulating leukocyte recruitment [21]. The primary receptor for *S100A8/9* is toll‐like receptor 4 (*TLR4*) and its activation leads to the release of massive pro‐inflammatory cytokines to exacerbate inflammation (Figure 3G) [21, 22].*TLR4* expression was significantly increased in coinfected patients with severe disease compared to those with mild symptoms and healthy donors (Figure 3G), particularly in inflammatory monocytes (Mono_CD14) (Figure S6E,F, Supporting Information). *S100A8/9*‐*TLR4* signaling triggers the *MyD88*‐dependent pathway by recruiting and activating IRAKs (e.g. *IRAK1*, *IRAK3*, and *IRAK4*) and *TRAF6*, amplifying the inflammatory response and causing severe tissues damage (Figure 3G) [21]. The expression of these key genes in the *S100A8/9‐TLR4*‐*MyD88* signaling pathway were also significantly increased in the severe coinfected group, especially for inflammatory monocytes (Figure 3G, Figure S6F, Supporting Information). These findings highlight that coinfected patients with severe disease displayed *S100A8/9‐TLR4*‐inflammatory characteristics, indicating the importance of *S100A8/9* for designing effective therapeutic methods to alleviate immunopathogenesis in severe patients. In addition to *S100A8/9*, other S100 proteins (e.g. *S100A6/11/12*) can also trigger the *TLR4*‐*MyD88*‐dependent pathway [23, 24], potentially exacerbating *S100A8/9*‐driven inflammation in the severe coinfected group (Figure 3D, Figure S6C,D, Supporting Information).

Inflammatory storm may be associated with cellular cross‐talk among inflammatory cells through the release of various cytokines [1]. We thus examined the ligand‐receptor pairing patterns of the four inflammatory cell clusters from the severe group (Figure S6G, Supporting Information). Notable ligand‐receptor interactions among the four inflammatory subsets were observed (Figure S6G, Supporting Information). The major inflammatory cell subset (Mono_CD14) expressed multiple receptors (e.g. *TNFRSF1A, DPP4, TNFRSF10B, TNFRSF1B*, and *IL15RA*) (Figure S6G, Supporting Information, Figure 3H), indicating that this cell subset can concurrently respond to multiple cytokines released from other cells. In addition, our analysis found that the interactions between classical monocytes and other inflammatory cells may mainly rely on TNF|TNFRSF1A, TNFSF13|TNFRSF1A, IL1B|IL1RN, TNFSF13B|HLA‐DPB1, CXCL2|DPP4 etc. (Figure S6G, Supporting Information, Figure 3H). Taken together, we observed the potential molecular basis for interactions between inflammatory cells in severe coinfected patients.

In addition to the top 5 cytokines, we also measured the plasma concentrations of 30 cytokines in coinfected patients, and observed a significant increase in various pro‐inflammatory cytokines (e.g. IL‐1B, IL‐6, IL‐10, IL‐8, IP‐10, IL‐15, IL‐1RA, MIP‐1α/β, MCP‐1 etc.) in coinfected patients, including those in the severe group (Figure S71, Supporting Information). However, these pro‐inflammatory cytokines were not detected in the PBMCs of coinfected patients (Figure 3), suggesting that the source of serum pro‐inflammatory cytokines is from the primary respiratory infection site.

### 2.4 Dysregulated T Cell Responses in Severe Coinfected Patients

**FIGURE 4:**
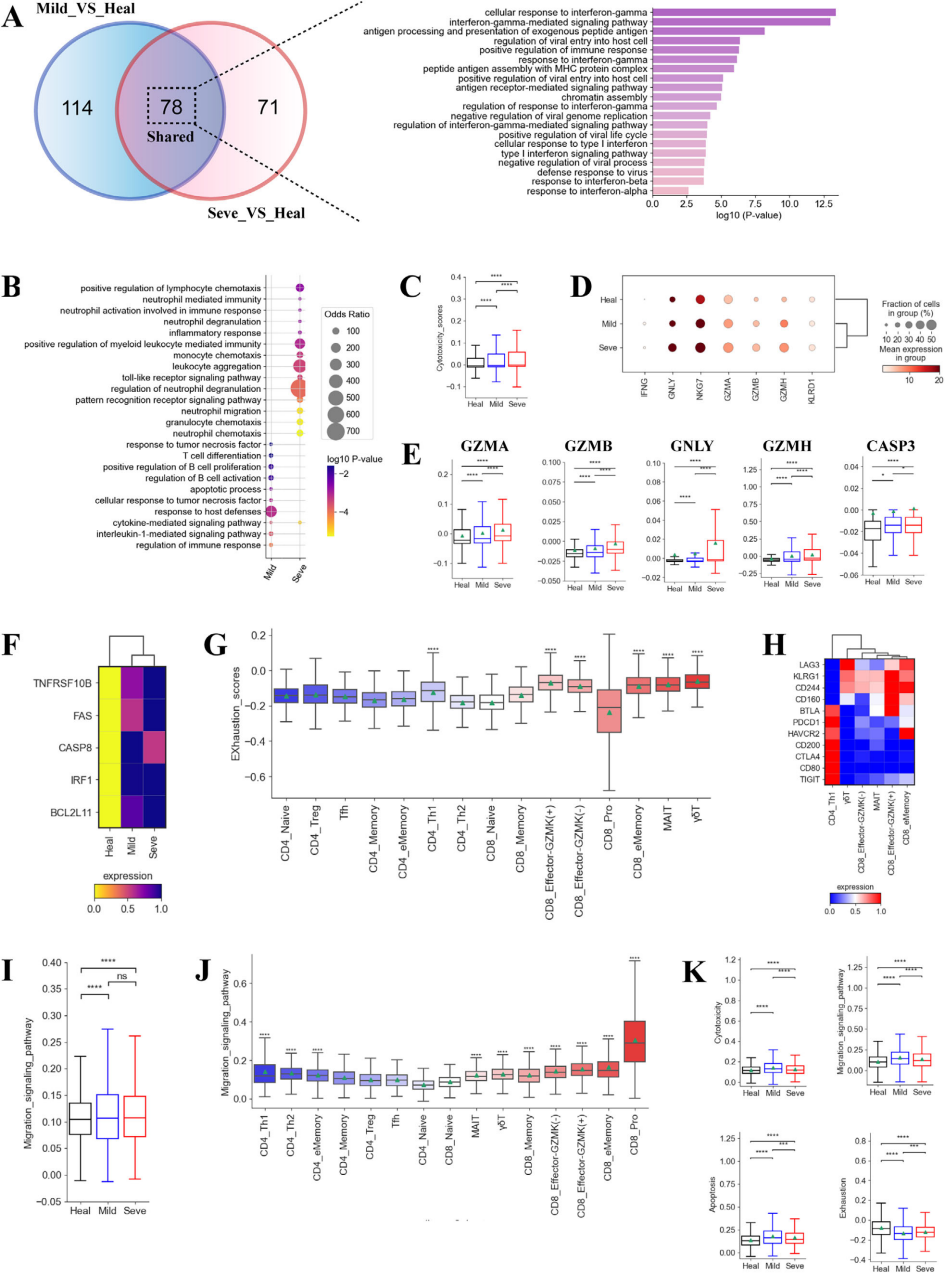
Expression differences in T cells across TB and COVID‐19 coinfected groups. (A) Venn diagram illustrating number of upregulated genes in T cells. (B) Enriched GO biological process terms for upregulated genes in T cells. Only select terms are shown. (C) Box plots illustrating T cell cytotoxicity scores from different groups. (D) Dot plots of select genes in T cells from different groups. (E) Box plots of select genes in T cells from different groups. (F) Heatmap showing selected normalized expression of apoptosis‐associated genes in T cells from different groups. (G) Boxplots of the exhaustion scores of 15 T cell subtypes. Wilcoxon rank‐sum test was used to assess significance. (H) Heatmap showing normalized expression for selected T cell exhaustion‐associated genes for different groups. (I) Box plots of T cell cell migration scores from different groups. (J) Boxplots of the migration scores of 15 T cell subtypes. Wilcoxon rank‐sum test was used to assess significance. (K) Box plots of cytotoxicity, migration, apoptosis and exhaustion scores in NK cells from different groups.

Transcriptome analysis of T cells was conducted in coinfected patients. Compared to healthy controls, we found 192 and 149 upregulated differentially expressed genes (DEGs) in the mild and severe coinfected groups, respectively, of which, 78 were common (Figure 4A, Table S4, Supporting Information). GO (gene ontology) analyses found that these shared upregulated genes belonged to ‘interferon‐gamma response’, ‘type I interferon response’, consistent with the concepts that IFN‐γ and IFN‐I responses are essential to response caused by TB and viral infection, respectively (Figure 4A, Figure S7A, Supporting Information). Also enriched were genes for ‘defense response to virus’, consistent with an immune response against the COVID‐19 virus (Figure 4A). GO terms involved in ‘neutrophil chemotaxis, degranulation and activation’ and ‘inflammatory response’ were also enriched in coinfected patients with severe disease (Figure 4B), suggesting that T cells from severe patients may acquire the inflammatory features. These genes which are associated with inflammatory response (e.g. *S100A8/9/12*), were upregulated in the severe group compared to the healthy and mild groups.

We then analyzed the cytotoxic score of T cells in different groups. At the bulk level, T cells showed a higher cytotoxic state in coinfected patients with the severe group being the highest (Figure 4C). Further analysis revealed that several T cell subsets, including CD8_Memory, CD8_eMemory, MAIT and γδ T cells, displayed higher cytotoxic state in the severe group (Figure S8C, Supporting Information), suggesting that they may be major contributors of cytolytic‐associated immunopathology in severe patients. The coinfected patients, especially in the severe group, had multiple highly expressed cytotoxic genes, including *IFNG*, *NKG7*, *GNLY*, GZMA, *GZMH, GZMB*, and *KLRD1* (Figure 4D). Although granzymes are important for killing pathogens or pathogen‐infected cells, their overexpression can cause immunopathology by damaging the extracellular matrix and eliciting inflammation. Therefore, increased expression of various T cell cytolytic proteins may be related to the immunopathology in coinfected patients, particular for those with severe disease.

We then investigated granzyme/perforin‐mediated apoptosis as effector proteins (e.g. *GZMA*, *GZMB*) can also cause cell apoptosis [25]. Perforin/granzyme pathway‐related genes, including *GZMA*, *GZMB*, *GZMK*, *GNLY*, and *CASP3*, were significantly upregulated in coinfected patients, with the highest expression found in severe patients (Figure 4E). In addition to perforin/granzyme related pathways, the *TNF*, *FAS*, and *IRF1* pathways [1] can also lead to apoptosis of T cells and thus were investigated (Figure 4F). Key genes in these pathways (e.g. *TNFSF10B*, *CASP8, FAS*, *IRF1*) were significantly elevated in coinfected patient (Figure 4F), especially in the severe group. These data suggest that the *TNF*‐, *FAS*‐, *IRF1*‐ perforin/granzyme‐apoptosis pathways may cause apoptosis of T cells. We found that CD4_Naïve, CD4_Th1, CD4_Treg, CD8_Naïve, CD8_Effector‐GZMK^(‐)^, CD8_Effector‐GZMK^(+)^, CD8_eMemory, MAIT, and γδT were more likely to undergo apoptosis in coinfected patients based on an apoptosis scoring system (Figure S8D, Supporting Information).

We next evaluated the exhaustion state of T cells in coinfected patients (Figure 4H). At the bulk level, a significant upregulation of exhaustion score was observed in the severe coinfected group compared to the mild and healthy groups, suggesting that T cells were more likely to experience exhaustion in severe patients (Figure S7E, Supporting Information). The exhaustion score for six T cell sub‐clusters (CD4_Th1, CD8_Effector‐GZMK^(+)^, CD8_Effector‐GZMK^(‐)^, CD8_eMemory, MAIT, and γδT) were significantly higher, indicating that these subtypes may be important exhaustion T cells (Figure 4G). Consistently, exhausted Th1 cells have also been reported in severe TB patients [1]. For each exhaustion cell subtype, we identified unique gene expression patterns for signature exhaustion genes (Figure 4H), potentially suggesting various mechanisms causing exhaustion in severe patients. High expression of various inhibitory molecules (*PD1*, *CD200*, *HAVCR2*, *CTLA4*, *CD80*, and *TIGIT*) were observed in exhausted T cells (Figure 4H). These inhibitory molecules interact with their receptors (e.g. *PD1* with *PDL‐1*/*PDL‐2*, and *HAVCR2* with galectin‐9), to recruit tyrosine phosphatases *SHP1/SHP2*, resulting in reduced cellular proliferation and cytokine secretion. Consistently, we found significant upregulation of *SHP1* and *SHP2* in the severe coinfected group (Figure S8F, Supporting Information). We also observed upregulation of *PRDM1* in coinfected patients with severe disease (Figure S8F, Supporting Information). It has been documented that elevated *PRDM1* is related to upregulated expression of inhibitory receptors and decreased poly functionality in exhausted cells. Our findings suggest that exhausted T cells, especially for activated CD4^+^ T (e.g. CD4_Th1) and effector CD8 + T cells (e.g. CD8_Effector‐GZMK^(+)^), may have a key role in causing immune dysfunction in severe coinfected patients.

Using a migration scoring system, we investigated the T cell migration state in coinfected groups (Figure 4I,J). We observed T cells from coinfected groups exhibited a stronger migration status compared to healthy donor but no difference was seen between severe and mild patients (Figure 4I). Ten T cell subclusters, including CD4_Th1, CD8_Pro, MAIT etc., in coinfected patients likely underwent migration (Figure 4J). The migration‐related genes (*CXCL17*, *XCL2*, *CXCL13*, *CXCL2*, *CXCL8* etc.) were highly expressed in coinfected patients (Figure S8G, Supporting Information). The increased activation of T cell migration pathway indicates that T cell migration may be associated with a decrease in their population, especially for severe patients. Consistent with T cells, coinfected patients also highly expressed genes related with cytotoxicity, cell apoptosis and cell exhaustion in NK cells relative to healthy donors (Figure 4K). However, NK cells in coinfected did not appear to undergo exhaustion (Figure 4K).

### 2.5 B Cell Heterogeneity in Coinfected Patients

**FIGURE 5:**
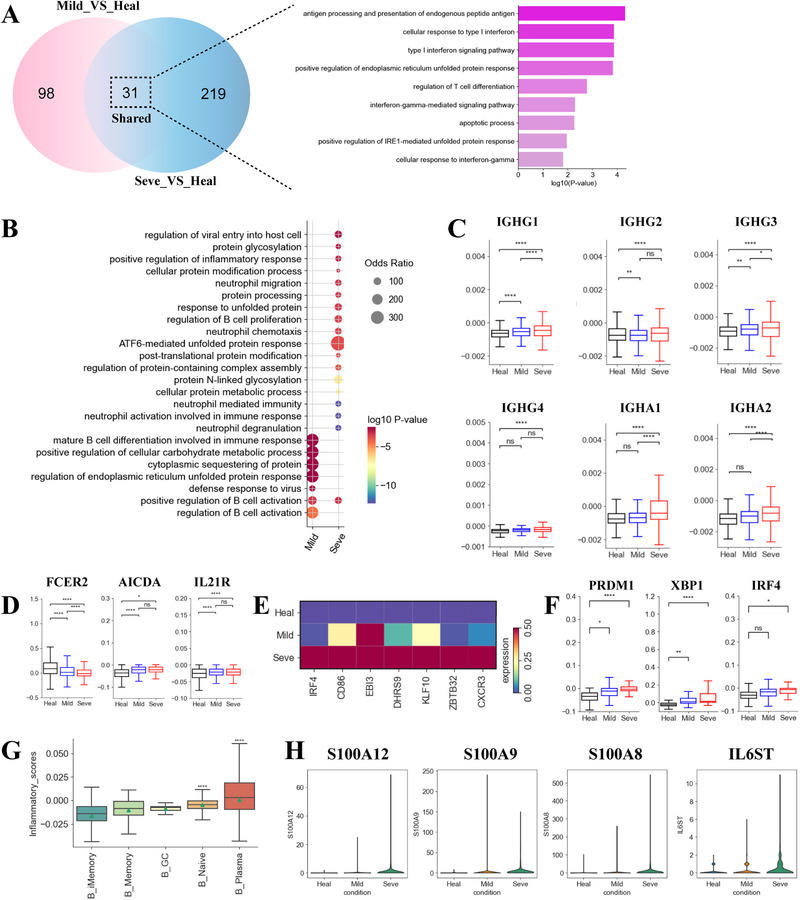
Expression differences in B cells across TB and COVID‐19 coinfected groups. (A) Venn diagram illustrating number of upregulated genes in B cells. (B) Enriched GO biological process terms for upregulated genes in B cells. Only select terms are shown. (C) Box plots of selected genes (e.g. IGHG1) in B cells between healthy and coinfected groups. (D) Box plots of selected genes related to activation of naïve B cells between healthy and coinfected groups. (E) Heatmap illustrating normalized expression for selected genes associated with activation of memory B cells between different groups. (F) Box plots of selected genes in plasma cells between healthy donors and coinfected patients. (G) Boxplots of the inflammatory scores of 5 B cell subtypes. Wilcoxon rank‐sum test was used to assess significance. (H) Violin plots of selected genes in B cells between healthy donors and coinfected patients.

Five B‐cell subsets displayed disease heterogeneity (Figures 2B,C and 5). Notably, the B_plasma population was elevated in severe cases, while the other four subsets were depleted in these patients (Figure 2, Figure S3, Supporting Information). This increase in B_plasma cells indicate the production of protective SARS‐CoV‐2 neutralizing antibodies. Functional analysis of upregulated DEGs in B cells from coinfected groups compared to controls (129 and 250 DEGs in mild and severe cases, respectively) (Figure 5A, Table S5, Supporting Information) revealed enrichment in pathways associated with protein complex assembly, transportation and modification‐associated pathways. This enrichment suggests that a large number of antibodies are being created (Figure 5A,B). Further analysis of IgA and IgG distribution revealed that coinfected patients had higher IgA and IgG compared to healthy donors (Figure 5C, Figure S9A, Supporting Information). Interestingly, severe patients had the highest IgA and IgG levels, implying elevated titers of SARS‐CoV‐2 antibodies in their serum (Figure 5C, Figure S9A, Supporting Information). This finding in coinfected patients with severe disease is consistent with previous observation that single‐infected severe COVID‐19 patients also had higher titers of antibodies.

In addition to plasma B cell expansion, pathways associated with B cell activation, differentiation, and proliferation were enriched in coinfected patients (Figure 5B). We next investigated the expression of key genes (e.g. *FCER2*, *PRDM1*) involved in B cell activation associated processes. Significant activation of naïve B cells was observed, characterized by FCER2 downregulation and *AICDA* and *IL21R* upregulation (Figure 5D) [1, 26]. Memory B cells in coinfected patients, particularly those with severe cases, highly expressed genes related to memory B cell activation (e.g. *IRF4*, *CD86*, and *EBI3* etc.) (Figure 5E) [27]. This expression pattern suggests potential memory B activation. In plasma cells, we examined the transcription factors *IRF4*, *XBP1*, and *PRDM1*, which determined plasma cell identity and function (e.g. *IRF4* regulates immunoglobulin class‐switch recombination, *XBP1* is important for increasing protein synthesis and *PRDM1* promotes immunoglobulin synthesis) [27, 28]. As expected, we observed upregulation of *IRF4*, *XBP1*, and *PRDM1* in the coinfected group, with the highest expression in the severe group (Figure 5F), further supporting the hypothesis that patients with severe symptom may have higher antibody titers. We also observed increased *CD2AP* expression on CD4^+^ T cells from coinfected individuals compared to healthy donors (Figure S9B, Supporting Information). *CD2AP* on CD4^+^ T cells is known to modulate Tfh cell differentiation and augment antiviral antibody responses for protection [27]. Additionally, *TNFSF14* expression, which supports plasma cell function, was upregulated in both CD4^+^ T and CD8^+^ T cells (Figure S9B, Supporting Information). *KDM5A*, which activates T and B cells, was also increased in CD8+ T cells (Figure S9B, Supporting Information) [27]. Our data collectively indicates that elevated plasma cells and upregulated B cell activation‐related genes in coinfected patients, particularly those with severe disease, may play a key in defensing against the virus.

Similar with our observation in T cells, genes involved in ‘IFN‐I response signaling’ pathway were enriched in B cell subsets (Figure 5A,B). Genes associated with IFN‐I response (e.g. *IFITM1*/*3*, *OASL*, *IFI6*) were elevated in the coinfected groups, particular in those from the mild group (Figure S9C, Supporting Information), implying that mild patients may mount a stronger IFN response and thus control the virus more effectively. Furthermore, pathways related to inflammatory response and neutrophil activation related pathways were particularly enriched in severe patients, indicating potential inflammatory features in B cells from this group (Figure 5B). By examining inflammatory signature genes, we observed different inflammatory status for each B‐cell subset, with higher inflammatory scores in B_Naïve and B_Plasma subsets (Figure 5G). Further analysis showed a significant increase in the expression of inflammatory signature genes in B_Naïve and B_Plasma subsets from severe patients (Figure S9D, Supporting Information). Consistently, genes (e.g. *S100A8/9/12*) involved in inflammatory response were upregulated in severe patients (Figure 5H). Our findings suggest that B cells from severe patients exhibited inflammatory features, which might contribute to exacerbated inflammation in this group. Finally, similar to our previous results in sole COVID‐19 patients, we observed downregulation of several HLA class II genes in both mild and severe coinfected groups (Figure S9E, Supporting Information), indicating a potential dysregulation in the crosstalk between adaptive immune cells.

### 2.6 Remodeling of Myeloid Cells in Coinfected Patients

**FIGURE 6:**
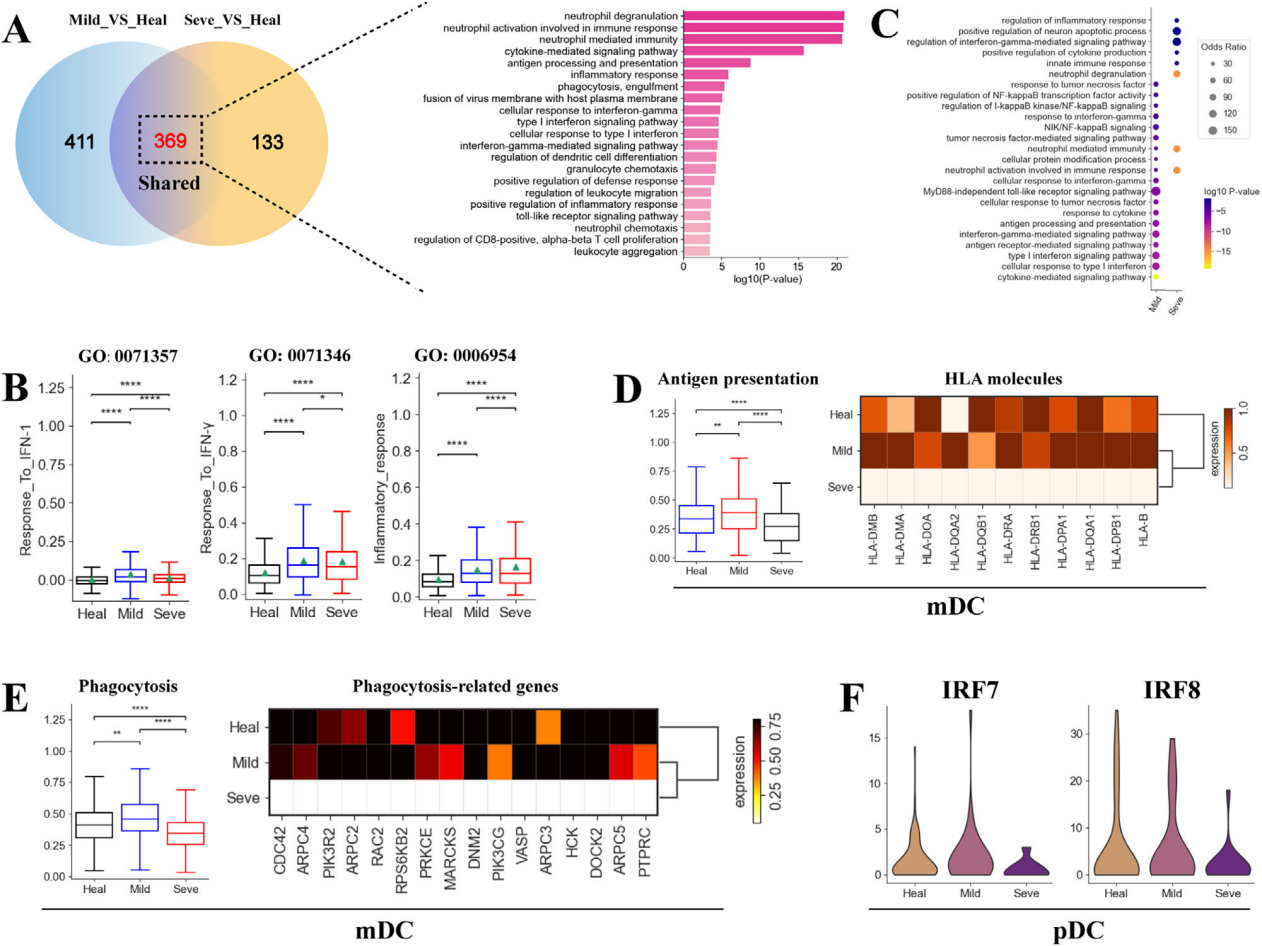
Expression differences in myeloid cells across TB and COVID‐19 coinfected groups. (A) Venn diagram illustrating number of upregulated genes in classical monocytes. (B) Box plots of three GO terms in classical monocytes across different conditions. (C) Enriched biological process terms for upregulated genes in classical monocytes. Only select terms are shown. (D) Box plots (Left) of antigen presentation scores in mDCs from different groups. Heatmap (Right) illustrating normalized expression for selected antigen presentation‐associated genes in mDCs from different groups. (E) Box plots (Left) of phagocytosis scores in mDCs across different conditions. Heatmap (Right) showing normalized expression for selected phagocytosis‐associated genes in mDCs from different groups. (F) Violin plots for selected genes in pDCs between healthy donors and coinfected patients.

Myeloid cells were clustered into 8 cell subtypes belonging to three common lineages: monocytes, megakaryocytes and DCs (Figures 1 and 2). We first focused on classical monocytes (Mono_CD14), because this subset represents the major peripheral myeloid cells (Figure S10A, Supporting Information) and plays a central role in triggering cytokine storms in the severe group (Figure 3). Strikingly, the Mono_CD14 population were significantly elevated in the coinfected group, with the most significant increase observed in severe group (Figure S10B, Supporting Information). We further compared the transcriptional features between Mono_CD14 from the mild/severe groups and healthy controls. We identified 800 and 502 upregulated genes in the mild and severe groups, respectively (Figure 6A, Table S6, Supporting Information). Among these DEGs, 369 upregulated genes were shared by both patient groups and were primarily involved in pathways associated with neutrophil mediated immunity, IFN response and inflammatory response (Figure 6A). Consistent with our GO analysis, Mono_CD14 cells from coinfected patients were characterized by elevated expression of various ISGs (e.g. *IRF1*, *IFITM2/3*) and inflammatory genes (e.g. *S100A8/9/11/12*) (Figure S10C, Supporting Information). Notably, the mild group displayed the highest expression of IFN response (Figure 6B), including those encoding IFN‐α/β and IFN‐γ. This aligns with the established roles of IFN‐1 and IFN‐γ in controlling viral infections and TB, respectively, suggesting that IFN response may influence disease severity in coinfected patients. In addition, signaling pathways associated with antiviral and anti‐TB response, such as ‘NIK/NF‐κB signaling’, ‘positive regulation of NF‐κB transcription factor activity’, ‘TNF‐mediated signaling pathway’ etc. were also upregulated in the mild group (Figure 6C). In contrast, the severe group exhibited the highest inflammatory response (Figure 6B), implying that a hyperinflammatory state may contribute to disease progression in coinfected patients.

Megakaryocytes (Mega) play a crucial role in hemostasis, but their contribution to coinfection pathogenesis remains largely unknown. We observed no significant upregulation of two key GO pathways (GO:0030168, GO: 0070527) associated with platelet aggregation and activation in coinfected patients (Figure S10D, Supporting Information), implying a low risk of thrombosis in these patients. While megakaryocytes are typically involved in the inflammatory response, we did not observe upregulation of pro‐inflammatory cytokines in these cells from coinfected patients, including those with severe disease (Figure S10D, Supporting Information). This finding aligns with our previous observation (Figure S3, Supporting Information) that megakaryocytes paly a relatively minor role in driving cytokine storms.

Classical dendritic cells (mDCs) are specialized antigen‐presenting cells that engulf pathogens, forming an important interface between innate and adaptive immunity [1]. Hence, we examined the phagocytosis and antigen presentation capacity of mDCs after SARS‐CoV‐2 and *Mtb* infection. mDCs from mild patients showed a significantly higher antigen presentation and phagocytosis capacity than healthy donors, but were lower in severe patients (Figure 6D,E). Accordingly, HLA molecules, especially class II molecules (e.g. *HLA‐DMA*, *HLA‐DMB*), were upregulated in mild patients but downregulated in the severe group (Figure 6D,E). Similarly, the expression of phagocytosis‐associated genes (e.g. *CDC42*, *PKI3R2*, *RPS6KB2* etc.) were increased in mild patients and decreased in severe patients. In addition, TFs (transcription factors) that regulate mDC development and function (e.g. *IRF4*, *RBPJ*, and *RELB*) exhibited lower expression levels in severe patients (Figure S10F, Supporting Information). The downregulation of these key TFs, coupled with reduced antigen presentation and phagocytosis capacity, indicates that mDCs in coinfected patients with severe disease may be in a state of immune paralysis.

pDCs (plasmacytoid DCs) produces type I IFNs in response to viral infection and are specialized in microbial sensing [1]. Thus, we investigated core genes (e.g. *IRF7/8*) involved in IFN induction and microbial sensing, focusing on *IRF7* and *IRF8*. *IRF7* regulates IFN production by pDCs, while *IRF8* controls pDCs development and functional modules. The expression of *IRF7/8* were decreased in coinfected patients with severe disease (Figure 6F). Consistently, other genes related to IFN production and response, including *TLR7*, *SLC15A4*, *DERL3*, *ISG15*, *IFITM2*, and *IFITM1*, were also downregulated in severe patients (Figure S10G, Supporting Information). These data indicate IFN production and microbial sensing in pDCs from severe cases might be dysfunctional. Next, we also investigated genes associated with the development and function of pDCs. *CD62L* (*SELL*), an adhesin involved in movement of pDCs to high endothelial venules (HEVs), and *NRP1*, involved in forming primary immune synapse with T cells and promoting T cell proliferation, were both decreased in pDCs from the severe group compared to healthy donors, pDCs had a lower expression of the two genes in severe patients (Figure S10H, Supporting Information). Additionally, *BCL11A*, which regulates pDC development and is an essential lineage‐specific factor, was also downregulated in the severe group (Figure S10H, Supporting Information). These findings indicate that pDC development and differentiation may be hindered in coinfected patients with severe disease.

## 3 Discussion

Both COVID‐19 and tuberculosis (TB) infect the lungs, disrupt the immune system function, and exhibit similar clinical manifestations. However, information regarding SARS‐CoV‐2 and (*Mycobacterium tuberculosis*) *Mtb* coinfections, including its potential impact on host cellular responses and pathogenesis, remains limited. This knowledge gap hinders the development of effective treatment methods, accurate disease prognosis predictions, and a comprehensive understanding of disease heterogeneity. In this study, we integrated clinical findings (Table S1, Supporting Information), laboratory examinations (Figure S1, Supporting Information), and scRNA‐Seq analysis to provide an integrated and detailed view of TB and COVID‐19 coinfection.

We first assessed alterations in the systemic immune response of coinfected patients at the single‐cell level. We identified nine main cell types and 31 cell subsets (Figures 1 and 2), providing insights into the cellular and molecular response to TB and COVID‐19 coinfection. Notably, immune cell composition was severely altered in coinfected patients with severe disease (Figure 1), particularly within the lymphocyte lineage. Lymphocytes, including CD4^+^T, CD8^+^T, B cells, NK cells, and innate lymphoid cells (e.g. MAIT, γδ T), were markedly depleted in severe coinfection, indicating that lymphopenia is a prominent characteristic in severe coinfections. While lymphopenia has been observed in sole TB or COVID‐19 severe patients [1, 29], our data indicates that severe coinfection has a more selective impact on B and innate lymphoid cells (Figure 1). This contrasts with T cell‐selective lymphopenia observed in COVID‐19 [29] and NK cell‐selective lymphopenia observed in TB [1]. The factors underlying the selective depletion of B cells and innate lymphoid cells in severe coinfection remain unclear and warrant further investigation.

Lymphopenia is a common feature of many respiratory viral infections (e.g. SARS‐CoV‐2, RSV, human rhinovirus and influenza virus etc.) and TB, particularly in patients with severe disease [29]. However, the mechanisms underlying lymphopenia in these infectious diseases remain incompletely understood. Some studies suggest a potential link between lymphopenia and elevated levels of IL‐10, TNF or IL‐6, which may act directly on lymphocytes or indirectly via other cell lineages (e.g. neutrophils and DCs) [30]. Consistent with this, we observed increased levels of IL‐6, IL‐10, and TNF in coinfected cases, especially those with severe disease (Figure S7, Supporting Information). Another possiblilty is that that the observed peripheral lymphopenia reflects the recruitment of lymphocytes (e.g. B, NK, T cells) to the respiratory tract. Unfortunately, we did not collect the bronchoalveolar lavage fluid or lung tissue from coinfected patients, so it remains unknown if lymphopenia is also due to tissue infiltration. In addition, the activation of various apoptosis‐associated pathways (e.g. FAS‐induced apoptosis pathway) may also contribute to lymphocyte depletion [1]. In line with this, we observed elevated expression of pro‐apoptotic molecules (e.g. *CASP3*, *FAS*, *CASP8*, *IRF1* etc.) in coinfected patients with severe disease, suggesting that cell apoptosis may be associated with the lymphopenia observed in severe coinfection.

In contrast to decreased levels of lymphocytes in peripheral blood, we observed enrichment of myeloid cells (e.g. monocytes) in coinfected cases, particularly those with severe disease. Increased numbers of inflammatory cells have been hypothesized as one of the crucial etiologies driving severe disease progression in sole *Mtb* or SARS‐CoV‐2 infection [1]. Our findings confirm the presence of cytokine storms in severe coinfected patients, which may contribute significantly to disease progression and immunopathogenesis. Myeloid cells, particularly classical monocytes (Mono_CD14), were identified as a major source of the cytokine storm. While several pro‐inflammatory cytokines were notably elevated in severe coinfection, the S100 family proteins (*S100A8*/*A9*), mainly released by classical monocytes, may have a dominant role in causing inflammatory cytokine storms. Human S100 family proteins were elevated in various inflammatory cells (including monocytes), have been reported to be significantly increased in patients suffering from inflammatory diseases, including SARS‐CoV‐2 and *Mtb* infections [21, 24]. In our study, we observed significant upregulation of *S100A8*/*A9* in patients with severe coinfection compared to those with mild disease or healthy controls (Figure 3). Previous studies have validated that S100 family proteins (e.g. *S100A8/A9/A12*), acting as hyper‐inflammatory molecules with cytokine‐like properties, can trigger a pro‐inflammatory response via the *TLR4*‐*MyD88*‐signaling pathway [21, 22, 24]. As expected, we observed higher expression of key molecules involved in *TLR4*‐*MyD88*‐signaling pathway, including *TLR4*, *MyD88*, *IRAK3*, *TRAF6* etc., in severe patients than those with mild disease and healthy donors (Figure 3). These findings suggest that blocking S100 family molecules (*S100A8/A9/A12*) from binding to cell lineages expressing TLR4 (e.g. monocytes) might abrogate the *TLR4*‐*MyD88*‐signal and inhibit the pro‐inflammatory response. Therefore, our data highlight the potential of targeting the S100‐*TLR4*‐*MyD88*‐signaling pathway as a therapeutic strategy in severe coinfection. Specifically, anti‐S100 treatments, which aim to reduce the production of S100 family molecules, particularly S100A8/A9, may be beneficial in mitigating the inflammatory storm in patients with severe coinfection.

Within the monocyte population, we identified an MDSC subset (Mono_MDSCs), featured by elevated expression of neutrophil activation‐related genes (e.g. *S100A8/A9*) and reduced expression of MHC‐II molecules (Figure 2). MDSCs are a heterogeneous lineage of immature myeloid cells that expand under inflammatory conditions and are able to inhibit T cell response [1]. In our study, Mono_MDSCs was obviously enriched in patients with severe coinfection (Figure S3, Supporting Information). Based on the established immunosuppressive functions of MDSCs, we hypothesize that mono_MDSCs contribute to coinfection pathogenesis and disease progression by suppressing immune responses. This hypothesis is supported by recent reports describing similar findings in patients with severe COVID‐19 or TB alone [1, 10, 31]. In addition to Mono_MDSCs, we observed that both mDCs and pDCs also contributed to immune paralysis in coinfected patients with severe disease, as evidenced by the downregulation of key TFs, antigen presentation capacity and phagocytosis. Taken together, these findings suggest that functional dysregulation in myeloid cells during confection may contribute to increased disease severity.

Similar with our findings in myeloid cells, we also observed a dysregulated lymphocyte response in patients with severe coinfection, which further contributed to disease pathogenesis. The Th1 response, characterized by the release of cytokines such as IFN‐γ, plays an important role in controlling *Mtb* infection. However, in our study, patients with severe coinfection displayed lower levels of *IFNG* expression and a less pronounced Th1 gene signatures compared to those with mild disease. This suggest that a dysregulated Th1 response may contribute to ineffective *Mtb* immune responses in these cases. Moreover, multiple T cell subsets (e.g. CD4_Th1, MAIT, CD8_Effector‐GZMK^(+)^ etc.) showed markers of T cell exhaustion in patients with severe coinfection, which was evidenced by: (i) elevated inhibitory molecules such as *PDCD1*, *HAVCR2*, *LAG3* etc. and (ii) elevated exhaustion‐associated TFs (*PTPN6*, *PRDM1* etc.). T cell exhaustion is linked to inefficient control of various infections, including TB and COVID‐19. Hence, the presence of these exhausted T cell subsets might contribute to disease progression in coinfected patients. Furthermore, T cells from patients with severe coinfection showed a more cytotoxic phenotype compared to those from individuals with mild symptoms and healthy donors. Specifically, these cells highly expressed multiple cytotoxic proteins, including *GNLY*, *NKG7*, *GZMA*, *GZMB* etc. Previous studies have validated that these effector molecules can induce tissue damage, including in the lungs, by triggering inflammatory response and degrading the extracellular matrix. Therefore, we inferred that the high cytotoxic state of T cells may be related to immunopathology in severe coinfection. In line with this notion, we observed elevated expression of genes related to pro‐inflammatory responses and neutrophil‐mediated immunopathology (e.g. *S100A8/A9/A12*) in T cells from patients with severe coinfection, further supporting a role for these cells in disease pathogenesis.

B lymphocytes are also essential for antiviral responses, including against SARS‐CoV‐2 infection. Similar to T cells, B lymphocytes from patients with severe coinfection also highly expressed genes involved in inflammatory response and neutrophil‐mediated immunopathology, further contributing to the immunopathogenesis seen in severe coinfection. We observed an expansion of plasma cells (B_Plasma) and elevated levels of SARS‐CoV‐2‐specific IgG and IgA antibodies in severe coinfected patients, consistent with our previous findings in patients with COVID‐19 alone [32]. However, despite these robust antibody responses, these individuals progressed to severe disease, indicating that humoral immune response alone might not be sufficient to prevent disease progression in the context of coinfection. Collectively, our results reveal a dysregulated lymphocyte response in patients with severe coinfection that likely contributes to disease severity.

While our study provides a comprehensive single‐cell analysis of the immune landscape in TB and COVID‐19 coinfected patients, it is important to acknowledge some limitations: (i) The study included a relatively small number of patients, particularly in the severe coinfection group (*n* = 3). This small sample size limits the statistical power and generalizability of the findings. Future studies with larger cohorts are crucial for validating these findings; (ii) Our study primarily focused on PBMCs. Analyzing bronchoalveolar lavage fluid or lung tissue samples could provide more direct insights into the immune responses at the primary site of infection, potentially revealing localized immune responses not captured in the peripheral blood; (iii) Our study focused on peripheral blood immune cells, specifically excluding granulocytes due to technical limitations associated with the 10× Genomics platform. Consequently, any inferences regarding granulocyte involvement in our findings should be interpreted with caution.

In summary, this study provides a comprehensive single‐cell transcriptomic landscape of immune responses in TB and COVID‐19 coinfection. Our results reveal distinct molecular and cellular immune signatures associated with coinfection and uncover a state of systemic immune dysregulation in severe cases. These findings provide valuable insights into immunopathogenesis of TB and COVID‐19 coinfection and may inform the development of novel therapeutic strategies for this patient population.

## 4 Methods

### 4.1 Ethical Approval

Ethics for this study was approved by the Beijing Chest Hospital ethics committee (Ethical approval no. No. BJXK‐KY‐2023‐01). Each participant provided written informed consent.

### 4.2 Study Design and Participants

Eleven adults with COVID‐19 and TB coinfection were prospectively recruited and sampled during December 9, 2022 and January 10, 2023 in a TB designated hospital: Beijing Chest Hospital (Beijing, China). COVID‐19 cases were diagnosis by nasopharyngeal or throat swab using reverse transcription polymerase chain reaction (RT‐PCR) for SARS‐CoV‐2. TB cases were diagnosis by smear, culture or Xpert from sputum. The severe illness group consisted of patients admitted to ICU and required mechanical ventilation while the mild group were patients with no or mild pneumonia (Table S1, Supporting Information).

### 4.3 Single‐Cell RNA Sequencing

Fresh blood samples (*n =* 17) were collected and PBMCs were isolated as previously described [27, 28]. Briefly, the Countstar cell viability detection kit was used to determine cell viability, which was >90% for each sample. The 10X Genomics Chromium Controller Instrument, Chromium Single Cell 5′ library & gel bead kit v2(PN:000356)and Chromium Next GEM ChipK SingleCell Kit (PN:1000286) were used to generate scRNA‐Seq libraries according to manufacturer's instructions and sequenced on an Illumina Novaseq 6000 sequencer (2×150 bp) (Illumina, San Diego, CA) by NoveIBio Co., Ltd. (Shanghai, China).

### 4.4 Single‐Cell Transcriptomic Analysis

Single cell transcriptomic analysis was performed as previously reported [1, 16, 27, 28]. Briefly, Cell Ranger (V.7.1.0) with human reference (GRCh38) was used to acquire the raw gene expression matrices for each sample and then filtered with kallisto/bustools (kb v0.24.4). The matrix, filtered feature and barcode files were then further processed with anndata (ad) (v0.7.6) and Scanpy (sc) (v1.9.2) in python (v3.8.10) and finally merged into a single file with ad.concat [1, 27, 28].

Further filtering to remove low‐quality cells/doublets and gene expression normalization to 10,000 reads per cell were performed as previously described in Wang et al. [27, 28]. The top 1500 most highly‐variable genes (HVGs) between cells were then selected with sc.pp.highly_variable_genes as previously described. Dimension reduction with PCA (principal component analysis) to 20 PCA components was used to integrate different datasets and batch effect corrected for using Harmony algorithm [33] as described in Wang et al. [27, 28]. Finally, Harmony [34] was used to integrate the single‐cell data.

### 4.5 Cell Clustering and Annotations

Two rounds of unsupervised cell clustering with different resolutions, and based on neighborhood relations of cells, was performed with sc.tl.louvain. Nine major cells types were identified in the first round (Louvain resolution = 2.0) (CD8^+^ T cells, CD4^+^ T cells, mucosal‐associated invariant T cells (MAIT), gamma delta T cells (γδ T), B cells, NK cells (natural killer cells), monocytes, megakaryocytes, and dendritic cells (DCs)). CD8^+^ T, CD4^+^T, NK, B, monocytes and DC cells were sub‐divided in the second round (Louvain resolution 1.5) into different sub‐clusters. The signature genes for each cluster or sub‐cluster was identified with sc.tl.rank_genes_groups and then matched to canonical cell marker genes (Table S2, Supporting Information) for cell cluster/sub‐cluster annotation.

The proportion of each cell cluster/sub‐cluster was then calculated for different samples and disease types. R_O/E_ (ratio of observed versus expected cell numbers to eliminate technical variations on disease preference estimation) was used to calculate the disease preference for each cell type/subtype according to a previous report [1, 35].

### 4.6 Cell state Analysis of Cell Subtypes

After annotation, defined gene sets were used to compare the overall activation level and physiological activity of cell clusters/sub‐clusters. The inflammatory response and pro‐inflammatory cytokine genes were collected from published reports [1, 27, 28]. MsigDB was used to collect gene sets for response to type I interferon (GO:0034340), response to IFN‐γ (GO:0034341), inflammatory response (GO:0006954), platelet activation (GO:0030168), and platelet aggregation (GO:0070527). The cytotoxicity score, exhaustion score, phagocytosis score and antigen presentation scores were defined using 17, 11, 25, and 36 genes as listed in Table S7, Supporting Information [9] while the migration score was defined using genes from the leukocyte migration Pathway (GO:0050900). The cell state scores (average gene expression of predefined genes sets compared to reference genes) were calculated using sc.tl.score_genes. A student's t‐test was used to assess statistical significance of cell state scores between groups.

Trajectory analysis was conducted using PAGA in Scanpy (v1.5.1) with default parameters [36], and the analysis revealed a continuous cell type transition among the assigned discrete cell types. Differentially expressed genes (DEGs) were identified using the Scanpy function sc.tl.rank_genes_group with the parameter use_raw = True based on clusters or disease conditions (Wilcoxon rank sum test, adjusted *p* value <0.01, and fold‐change >1.5). Additionally, CellPhoneDB was employed with key parameters set to alpha = 0.01 and *p*‐value threshold = 0.01.

### 4.7 Plasma Cytokine Assays

As previously described [28], plasma levels for 30 cytokines (e.g. IL6, IL1β, IL8) were detected with the 34 plex Th1/Th2 human ProcartaPlex immunoassay (Thermo Fisher Scientific).

### 4.8 Statistics and Code Availability

Python and R were used to perform the statistical analysis and visualizations as described in the results, figure legends or methods above. The following symbols represent statistical significance in all figures: ns: *p* > 0.05; *: *p* ≤ 0.05; **: *p* ≤ 0.01; ***: *p* ≤ 0.001; ****: *p* ≤ 0.0001.

## Conflicts of Interest

The authors declare no conflicts of interest.

## Consent for Publication

Our manuscript dose not contains any individual person's data in any form.

## Transparency Declaration

The lead author and guarantor affirm that the manuscript is an honest, accurate, and transparent account of the study being reported; that no important aspects of the study have been omitted; and that any discrepancies from the study as planned and registered have been explained.

## Supporting information



Supporting Information

Supporting Information

Supporting Information

Supporting Information

Supporting Information

Supporting Information

Supporting Information

Supporting Information

Supporting Information

Supporting Information

Supporting Information

Supporting Information

Supporting Information

Supporting Information

Supporting Information

Supporting Information

Supporting Information

Supporting Information

## Data Availability

Experimental protocols and pipelines were performed according to the official 10X Genomics and Scanpy websites. The data that support the findings of this study are openly available in China National Center for Bioinformation at https://ngdc.cncb.ac.cn/omix/release/OMIX0066801; reference number OMIX006681. The data reported in this paper have been deposited in the OMIX, China National Center for Bioinformation/Beijing Institute of Genomics, Chinese Academy of Sciences (https://ngdc.cncb.ac.cn/omix; accession no. OMIX006681).
