# Supplementary material for: Single‐Cell Transcriptomic Analysis of the Immune Response to COVID‐19 and Tuberculosis Coinfection
Source: Exploration (Beijing). 2025 May 8;5(5):20240022. doi: 10.1002/EXP.20240022 (PMC12561472; doi:10.1002/EXP.20240022)
Supplement: Supplementary file 1 — Supporting Information [file EXP2-5-20240022-s016.docx]

**Supplementary**

**Single-cell transcriptomic analysis of the immune response to COVID-19 and tuberculosis coinfection**

Yi Wang ^1,2*, 🖂^, Maike Zheng ^3,*^, Yun Zhang ^3^ Yu Xue^4^, Sibo Long^3^, Chaohong Wang^3^, Qing Sun^3^, Jun Yan^3^, Yiheng Shi ^3^, Bin Yang ^3^, Shang Ma^3^ , Tiantian Zhang ^3^, Lei Cao ^3^, Yan Chen ^3^, Wenfu Ju ^3^, Jing Zhang ^4^, Yan Zhao ^3^, Mengqiu Gao ^5^, Laurence Don Wai Luu ^6,7 🖂^, Xinting Yang ^4,*, 🖂^ and Guirong Wang ^3, 🖂^

^1^ Experimental Research Center, Capital Center for Children's Health, Capital Medical University, Capital Institute of Pediatrics, Beijing, 100020, P.R. China.

^2^ Molecular Diagnostics Center, Capital Center for Children's Health, Capital Medical University, Beijing, 100020, P.R. China.

^3^ Department of Clinical Laboratory, Beijing Chest Hospital, Capital Medical University, Beijing Tuberculosis and Thoracic Tumor Institute, Beijing, 101149, P.R. China

^4^ Tuberculosis Department, Beijing Chest Hospital, Capital Medical University, Beijing, 101149, P.R. China

^5^ Department of Emergency, Beijing Chest Hospital, Capital Medical University, Beijing, 101149, P.R. China

^6^ School of Life Sciences, University of Technology Sydney, Sydney, 2007, Australia.

^7^ School of Biotechnology and Biomolecular Sciences, University of New South Wales, Sydney, 2052, Australia

**^*^** These authors contributed equally to this article

^🖂^ Correspondence:

Prof. Guirong Wang, [wangguirong1230@ccmu.edu.cn](mailto:wangguirong1230@ccmu.edu.cn)

Dr. Laurence Don Wai Luu, [laurence.luu@uts.edu.au](mailto:laurence.luu@uts.edu.au)

Prof. Xinting Yang, [yl-14t@163.com](mailto:yl-14t@163.com)

Prof. Yi Wang, [wildwolf0101@163.com](mailto:wildwolf0101@163.com) (Leading contact)

**Figure legend**

**Figure S1. Detailed data output and visualization of major cell types identified in single-cell transcriptional profiling of PBMCs from 17 samples, related to figure 1.**

A. Distribution of the percentage of mitochondrial transcripts per cell (left), unique molecular identifier (UMI) counts per cell (middle) and gene counts per cell (right) detected for cells in each disease group.

B. The UMAP projection of the 9 major cell type in each of the three conditions. Cells are colored by the 9 major cell types.

C. The UMAP projection of the PBMCs in each of the three conditions. Cells are colored by the 3 conditions.

D. Relative proportion of the 9 cell subtypes derived from Heal, Mild and Seve conditions.

E. The UMAP projection of the 17 individual samples. Cells are colored according to each individual.

F. Stacked boxplots showing the relative proportion of the 9 major cell types in each individual sample.

G. The composition of each immune cell type across 3 conditions. The y-axis shows the average percentage of each immune cell. Conditions are displayed in different colors on the *x* axis. Student’s T-test was applied to test the significance. *p<0.05, **p<0.01, ***p<0.001, ****p<0.0001, ^ns^p>0.05.

**Figure S2. Characteristics of the integrated dataset and selected markers for cell sets/subsets in different cell lineages, related to Figure 1.**

A, C, E,G, I. The clustering result of B (A), CD4^+^T (C), CD8^+^T (E), NK (G) and Myeloid (I) cell subsets. Each point represents one single cell, colored according to cell type.

B, D, F, H, J. Dot plots of selected marker genes (Rows) for cell subset (Columns) within each cell lineage, including B (B), CD4^+^T (D), CD8^+^T (F), NK (H) and Myeloid (J) cell subsets.

**Figure S3. Detailed data output and visualization of single-cell transcriptomic profiling of PBMCs from 17 samples, related to Figure 1 and 2.**

A. The UMAP projection of the 31 cell types in each of the three conditions. Cells are colored by the 31 cell types.

B. The UMAP projection of the PBMCs in each of the three conditions. Cells are colored by the 3 conditions.

C. Relative proportion of the 31 cell subtypes derived from Heal, Mild and Seve conditions.

D. The UMAP projection of the 17 individual samples. Cells are colored according to each individual.

E. Stacked boxplots showing the relative proportion of the 31 cell types in each individual sample.

F. The composition of each immune cell type across 3 conditions. The y-axis shows the average percentage of each immune cell. Conditions are displayed in different colors on the *x* axis. Student’s T-test was applied to test the significance. *p<0.05, **p<0.01, ***p<0.001, ****p<0.0001, ^ns^p>0.05.

**Figure S4. Comparison of different immune cell types among patient groups, related to Figure 2.**

A. UMAP plots of mean gene expression from Ig gene signatures in B cells, split by condition.

B. PAGA analysis of B cell pseudo-time: the associated cell type and the corresponding status are listed.

C. PAGA analysis of CD4^+^ T cell pseudo-time: the associated cell type and the corresponding status are listed.

D. Dot plots of IFNG expression across different samples.

E. UMAP plots of mean gene expression from Th2 gene signatures, split by condition.

F. PAGA analysis of monocyte cell pseudo-time: the associated cell type and the corresponding status are listed.

G. Dot plots of selected marker genes (Rows) for monocyte subsets (Columns) within each cell lineage.

**Figure S5. Details of hyper-inflammatory subtypes associated with cytokine storm in PBMCs, related to Figure 3**

A. Box plots showing the cytokine score and inflammatory score across conditions at the bulk level.

B. Box plots showing the cytokine score and inflammatory score across conditions for B cell subtypes.

C. Box plots showing the cytokine score and inflammatory score across conditions for CD4^+^T cell subtypes.

D. Box plots showing the cytokine score and inflammatory score across conditions for CD8^+^T cell subtypes.

E. Box plots showing the cytokine score and inflammatory score across conditions for NK cell subtypes.

F. Box plots showing the cytokine score and inflammatory score across conditions for innate T cell subtypes.

G. Box plots showing the cytokine score and inflammatory score across conditions for myeloid cell subtypes.

A-G, Student’s T-test was applied to test significance. *p<0.05, **p<0.01, ***p<0.001, ****p<0.0001, ^ns^p>0.05.

**Figure S6. Details of hyper-inflammatory subtypes associated with cytokine storm in PBMCs, related to Figure 3**

A. Bar plots showing the percentage of non-inflammatory cells and inflammatory cells across three conditions.

B. Heatmap of normalized expression for selected pro-inflammatory cytokine genes across different conditions.

C. Pie charts showing the relative percentage contribution of each inflammatory cell type to the *S100A6*-, *S100A11*-, *S100A12*-score.

D. Box plots of *S100A6*-, *S100A11*-, *S100A12*-score across different conditions.

E. Pie charts showing the relative percentage contribution of each cell type to the *TLR4*-score.

F. Dot plots of selected marker genes (Rows) for 31 subsets (Columns) within each cell lineage.

g. Dot plot showing the interactions among inflammatory cell types in coinfected patients with severe disease. P values are indicated by circle sizes as shown in the scale on the right.

A and D, Student’s T-test was used to test statistical significance. *p<0.05, **p<0.01, ***p<0.001, ****p<0.0001, ^ns^p>0.05.

**Figure S7. The levels of 30 cytokines in each group.**

Comparison of each condition was done using Student’s T-test. *p<0.05, **p<0.01, ***p<0.001, ****p<0.0001, ^ns^p>0.05.

**Figure S8. Characterization of gene expression differences in T cells across conditions, related to Figure 4**

A. Heatmap of normalized expression for selected genes related to IFN-γ response and IFN-I response across different conditions.

B. Heatmap of normalized expression for selected Neutrophil-mediated and inflammatory response genes across different conditions.

C. Box plots of cytotoxicity scores in selected T cell subtypes across different conditions.

D. Box plots of apoptosis scores in T cell subtypes across different conditions.

E. Box plots of exhaustion scores in T cells across different conditions.

F. Box plots of selected genes in T cells across different conditions.

G. Heatmap of normalized expression for selected genes across different conditions.

C, D, E and F, Student’s T-test was used to test statistical significance. *p<0.05, **p<0.01, ***p<0.001, ****p<0.0001, ^ns^p>0.05.

**Figure S9. Characterization of gene expression differences in B cells across conditions, related to Figure 5**

A. Box plots of selected genes in B cells across different conditions.

B. Box plots of selected genes in CD4^+^T and CD8^+^T cells across different conditions.

C. Heatmap of normalized expression for selected genes in B cells across different conditions.

D. Box plots of inflammatory score in Naïve_B and B_plasma cells across different conditions.

E. Dot plots of selected HLA-II genes in B cells across different conditions.

A, B and D, Student’s T-test was used to test statistical significance. *p<0.05, **p<0.01, ***p<0.001, ****p<0.0001, ^ns^p>0.05.

**Figure S0. Characterization of gene expression differences in myeloid cells across conditions, related to Figure 6**

A. Stacked boxplots showing the relative proportion of myeloid cell types in each condition.

B. The composition of Mono_CD14 cell type across 3 conditions. The y-axis shows the average percentage of Mono_CD14. Conditions are displayed in different colors on the *x* axis. Student’s T-test was applied to test the significance. *p<0.05, **p<0.01, ***p<0.001, ****p<0.0001, ^ns^p>0.05.

C. Heatmap of normalized expression for selected genes in Mono_CD14 across different conditions.

D. Box plots of the scores from selected GO pathways in megakaryocytes across different conditions. Student’s T-test was applied to test the significance. *p<0.05, **p<0.01, ***p<0.001, ****p<0.0001, ^ns^p>0.05.

E. Dot plots of selected inflammatory-related genes in megakaryocytes across conditions.

F. Violin plots of selected transcription factors in mDCs across conditions.

G-H. Violin plots of selected genes in pDCs across conditions.
