# Supplementary figures and images for: Single‐Cell Transcriptomic Analysis of the Immune Response to COVID‐19 and Tuberculosis Coinfection
Source: Exploration (Beijing). 2025 May 8;5(5):20240022. doi: 10.1002/EXP.20240022 (PMC12561472; doi:10.1002/EXP.20240022)

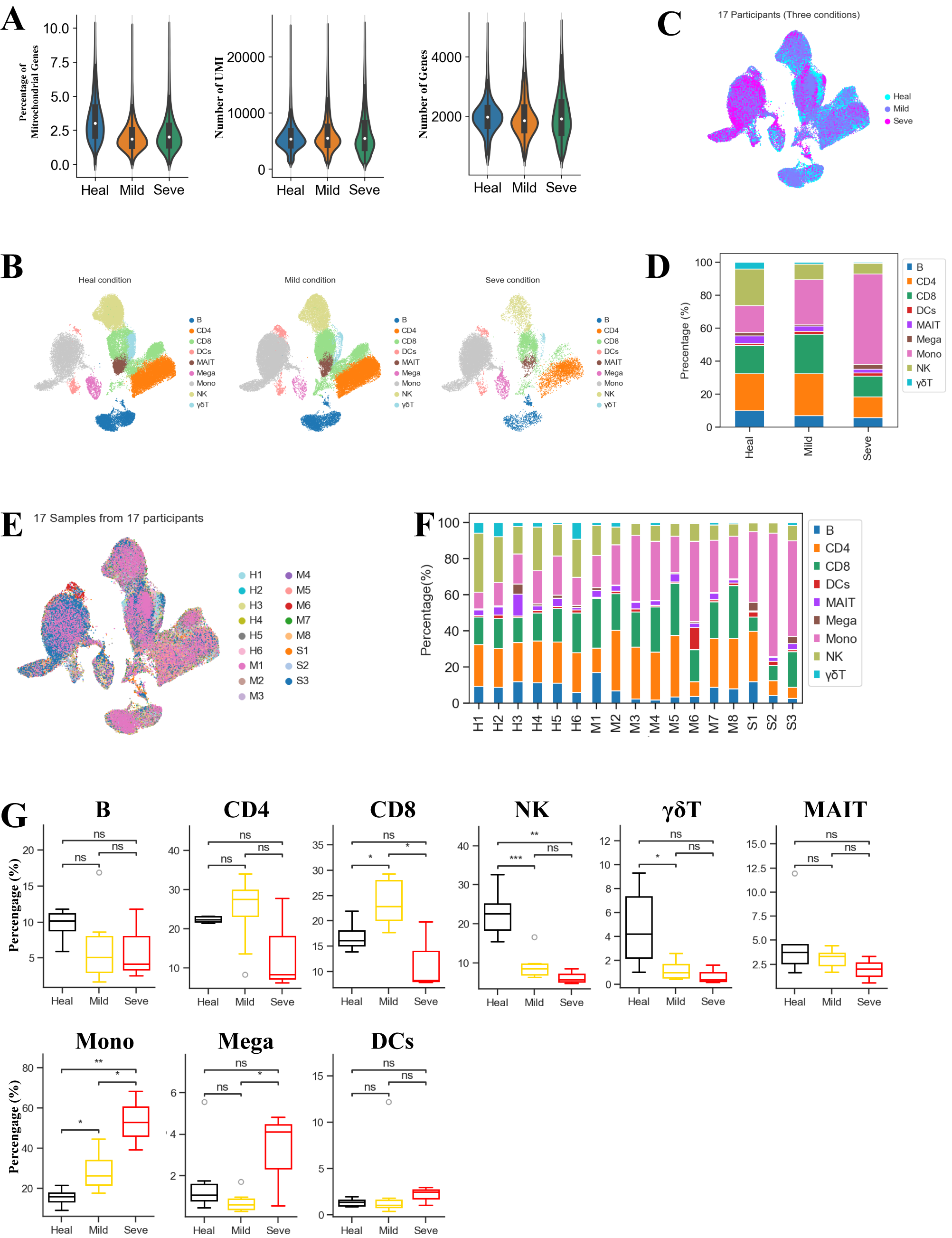

Supplement: Supplementary file 2 — Supporting Information [file EXP2-5-20240022-s010.tif]

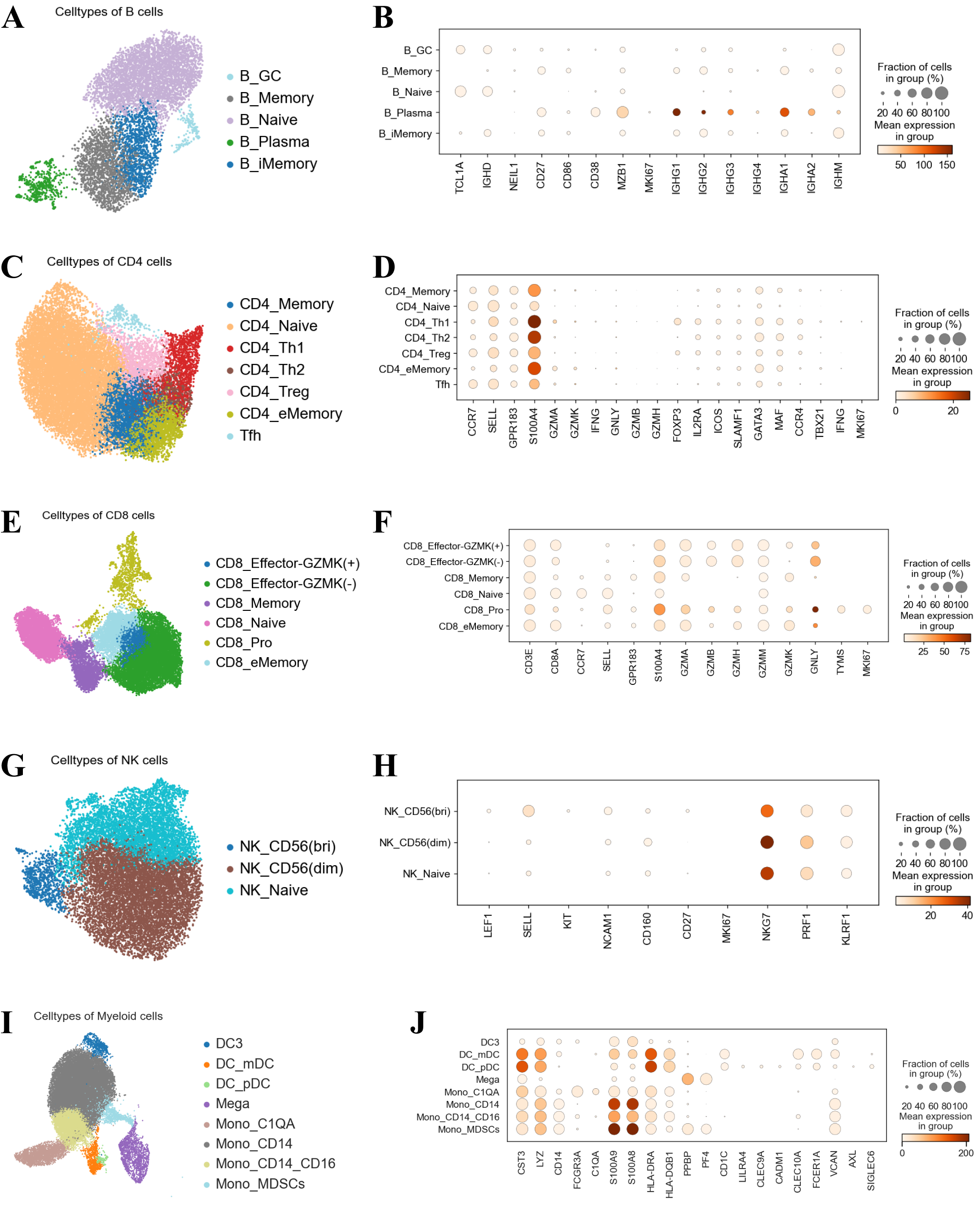

Supplement: Supplementary file 3 — Supporting Information [file EXP2-5-20240022-s005.tif]

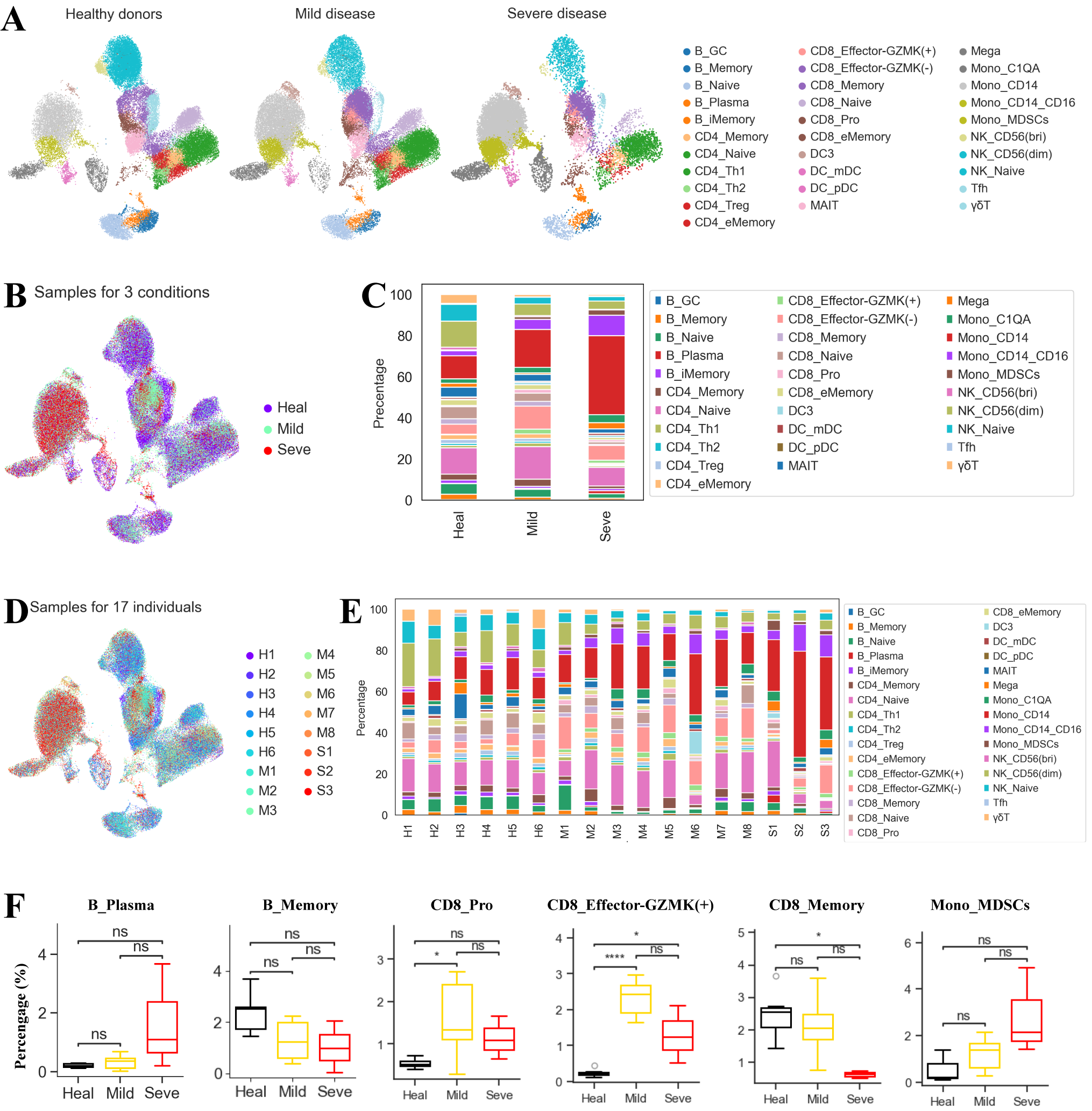

Supplement: Supplementary file 4 — Supporting Information [file EXP2-5-20240022-s004.tif]

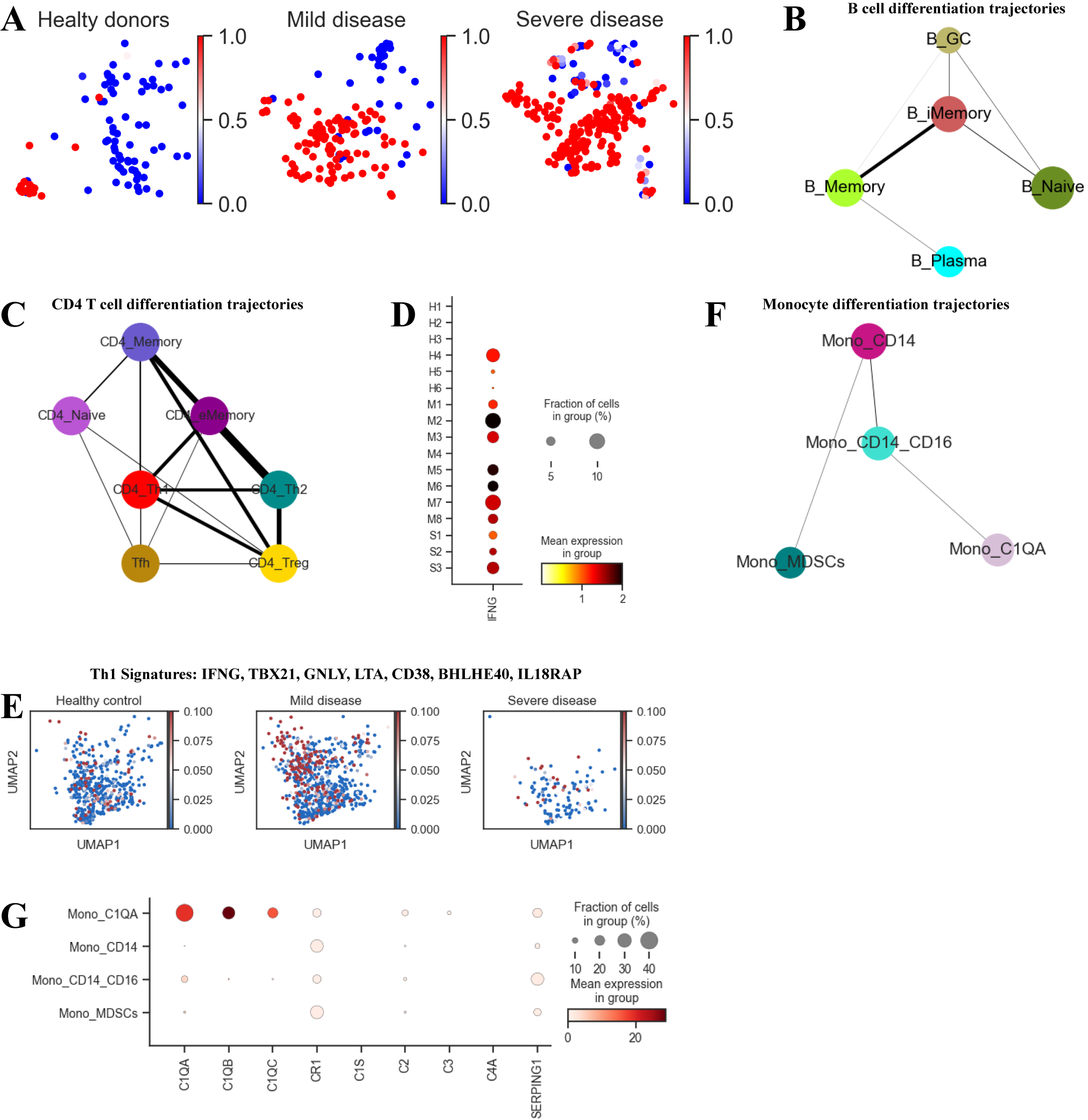

Supplement: Supplementary file 5 — Supporting Information [file EXP2-5-20240022-s013.tif]

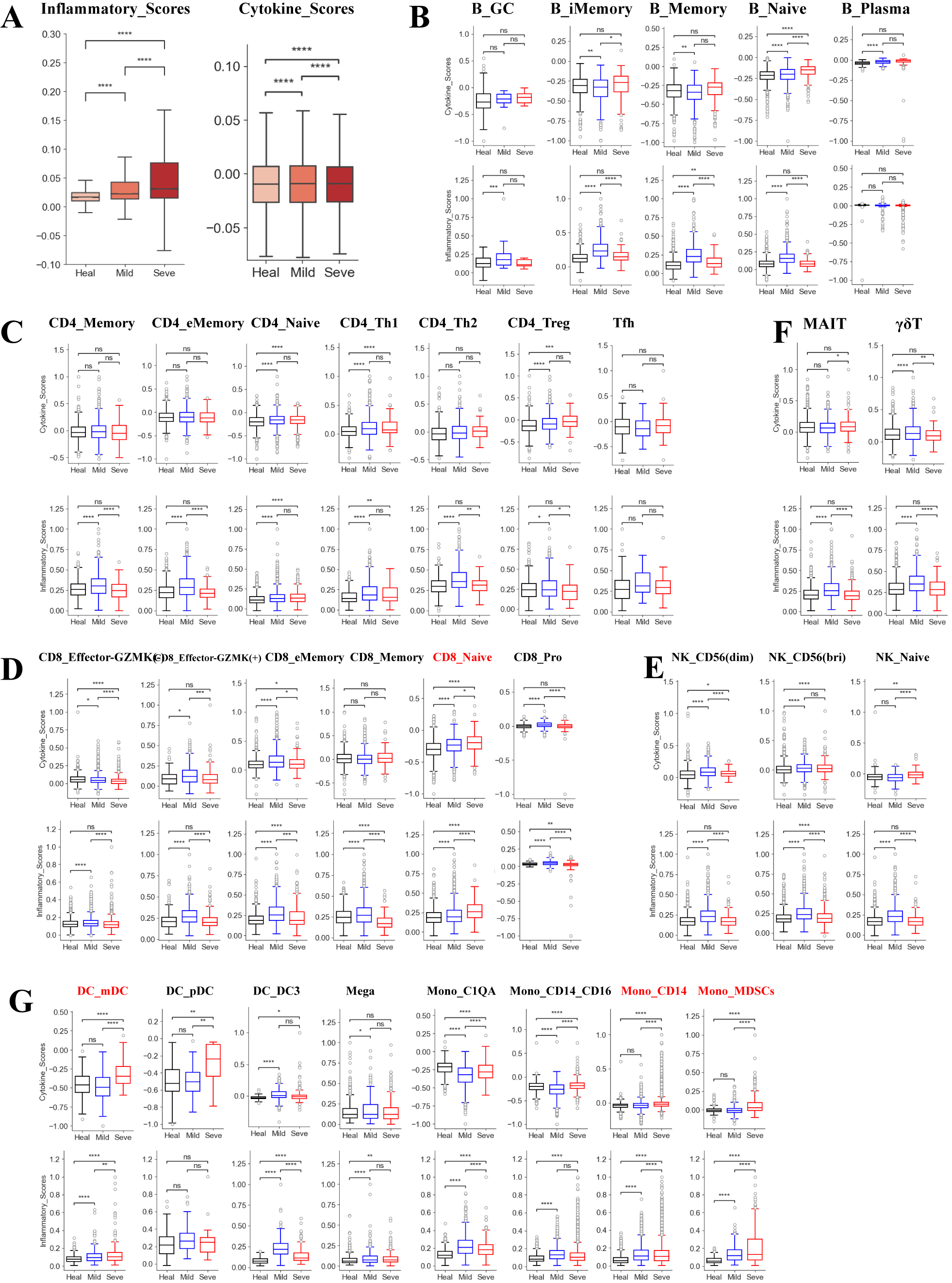

Supplement: Supplementary file 6 — Supporting Information [file EXP2-5-20240022-s007.tif]

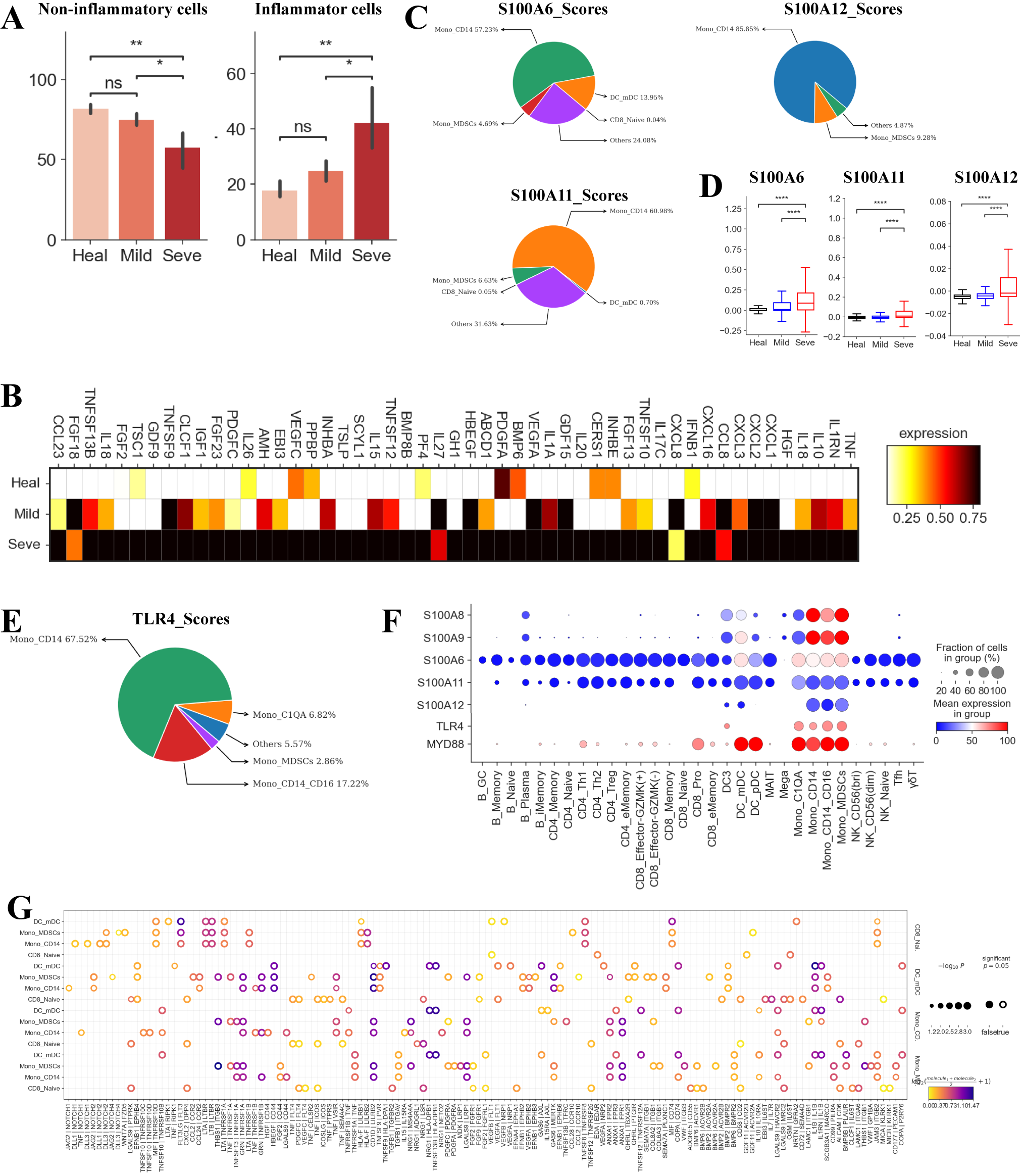

Supplement: Supplementary file 7 — Supporting Information [file EXP2-5-20240022-s017.tif]

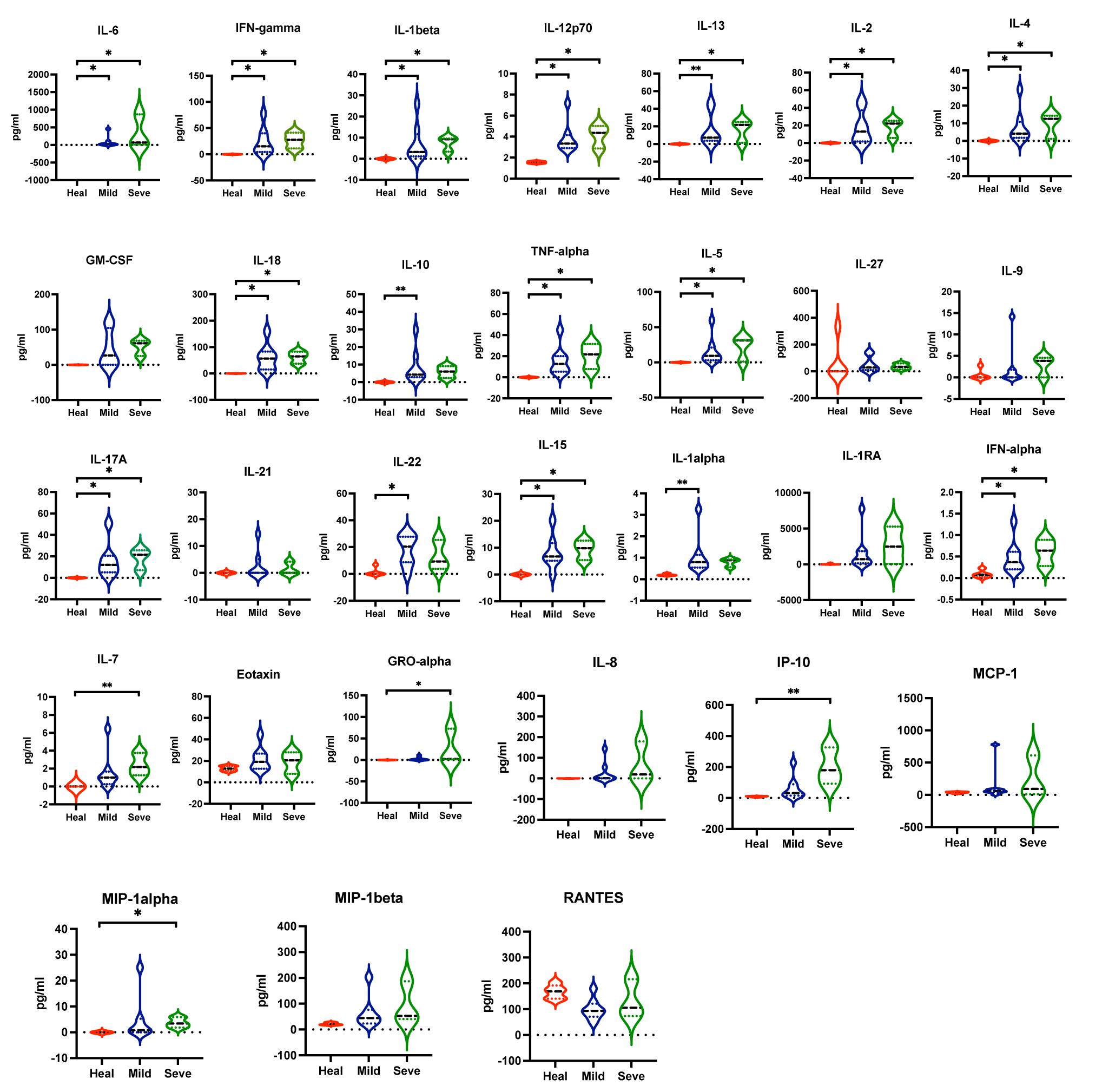

Supplement: Supplementary file 8 — Supporting Information [file EXP2-5-20240022-s015.tif]

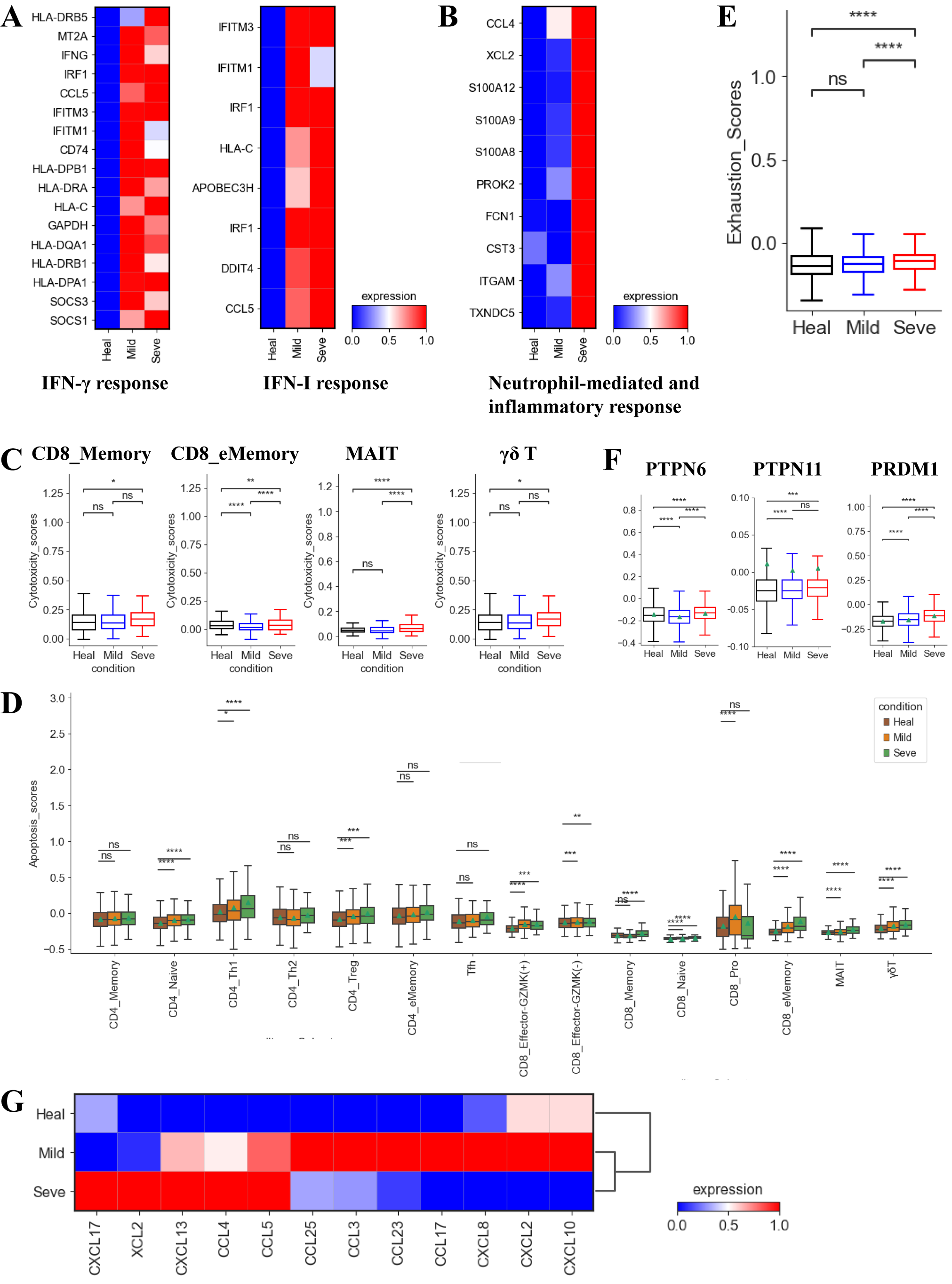

Supplement: Supplementary file 9 — Supporting Information [file EXP2-5-20240022-s008.tif]

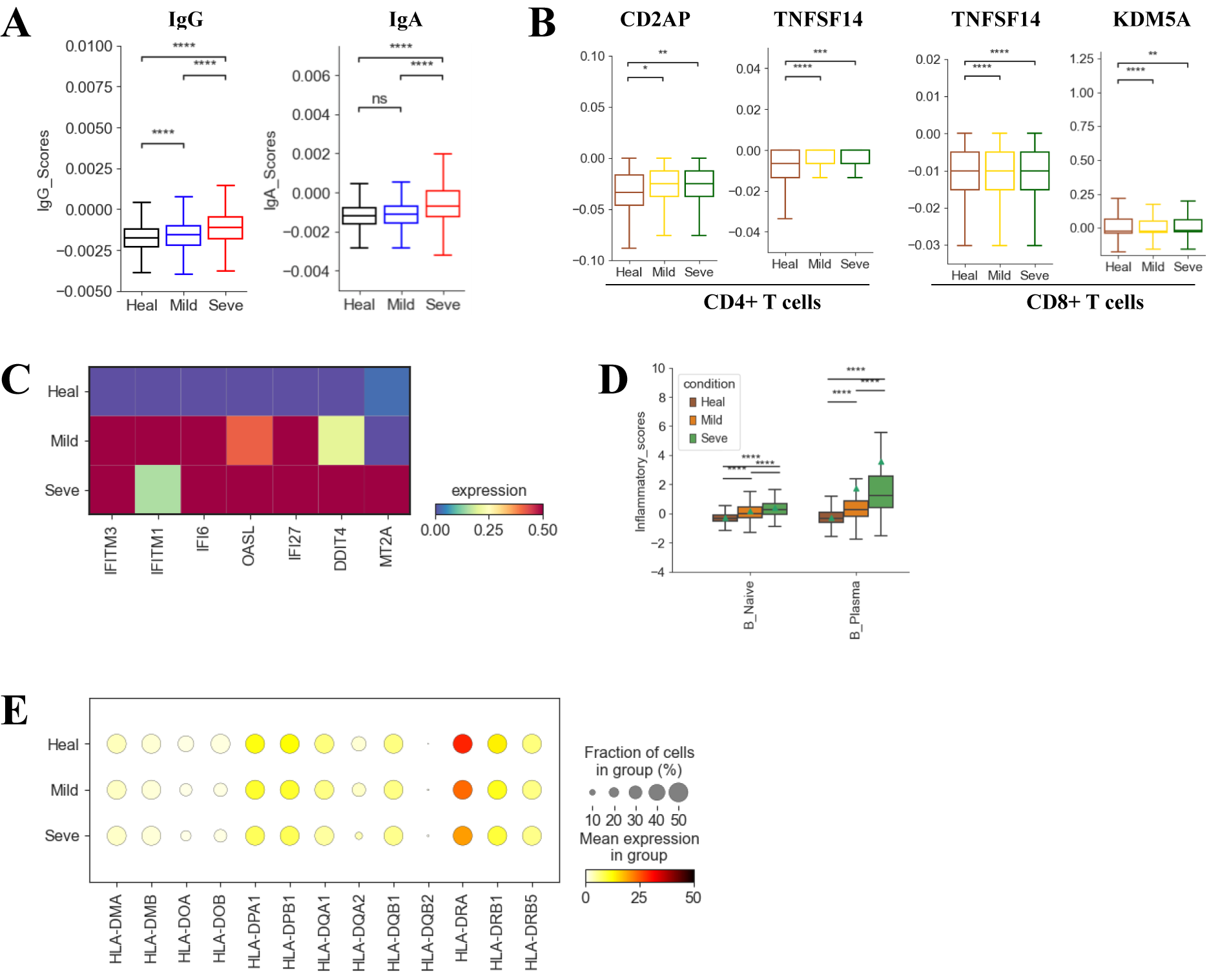

Supplement: Supplementary file 10 — Supporting Information [file EXP2-5-20240022-s018.tif]

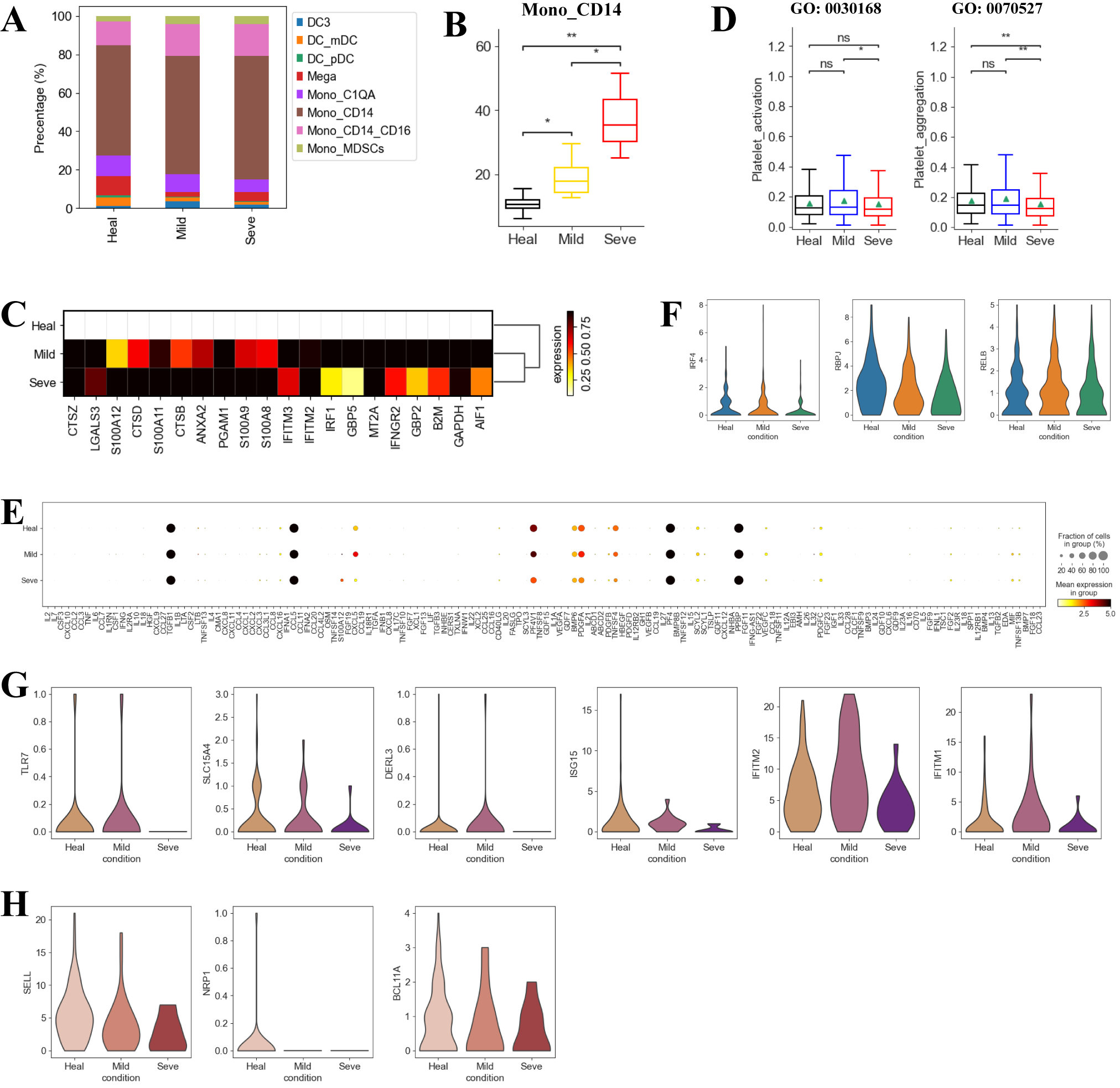

Supplement: Supplementary file 11 — Supporting Information [file EXP2-5-20240022-s001.tif]
