# Supplementary material for: Single‐Cell Transcriptomic Analysis of the Immune Response to COVID‐19 and Tuberculosis Coinfection
Source: Exploration (Beijing). 2025 May 8;5(5):20240022. doi: 10.1002/EXP.20240022 (PMC12561472; doi:10.1002/EXP.20240022)
Supplement: Supplementary file 12 — Supporting Information [file EXP2-5-20240022-s012.docx]

Supplementary Table 1. General characteristics and clinical features associated with paiticipents

| Healthy | | | | | | | | | |
| --- | --- | --- | --- | --- | --- | --- | --- | --- | --- |
| Chest X-ray or CT | 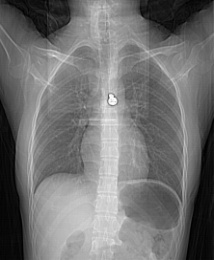 | 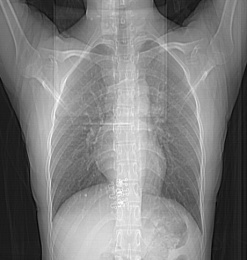 | 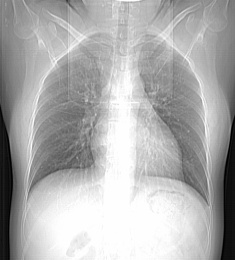 | 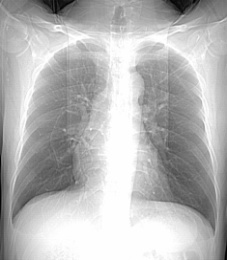 | 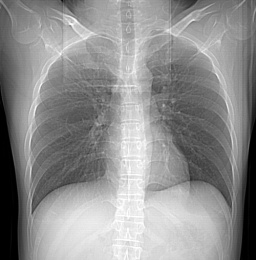 | 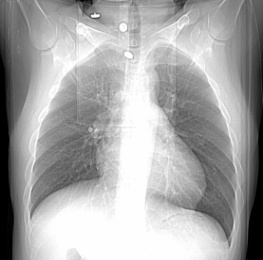 |  | |  |
| Code | H1 | H2 | H3 | H4 | H5 | H6 |  | |  |
| Gender | Female | Female | Female | Male | Male | Male |  | |  |
| Age (years) | 23 | 25 | 33 | 39 | 28 | 28 |  | |  |
| Mild | | | | | | | | | |
| Chest X-ray or CT | 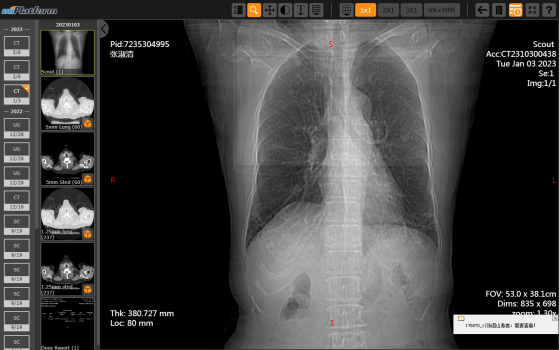 | 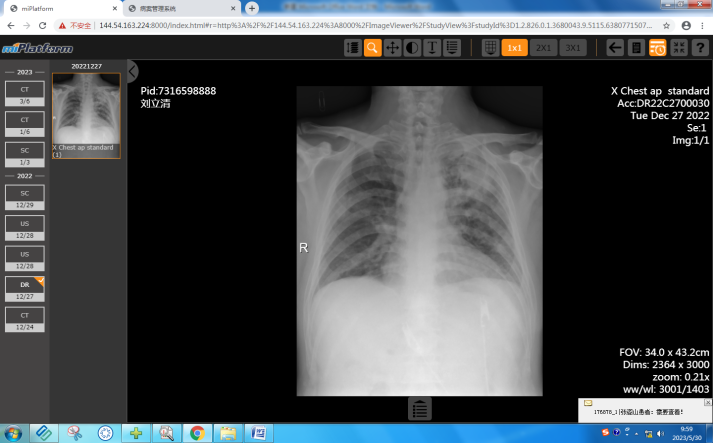 | 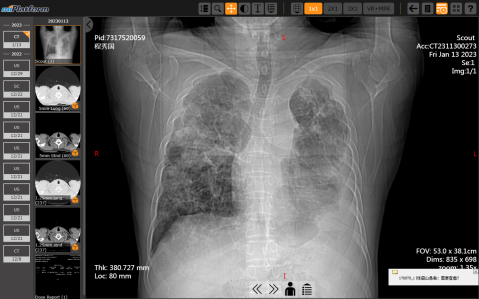 | 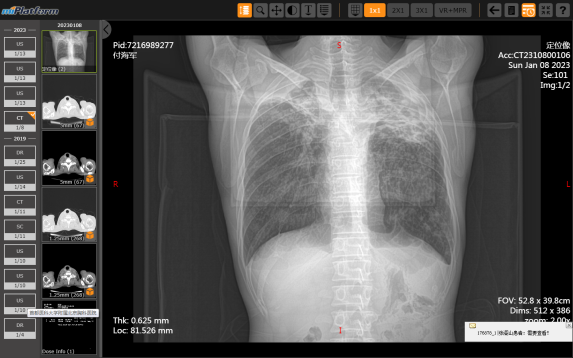 | 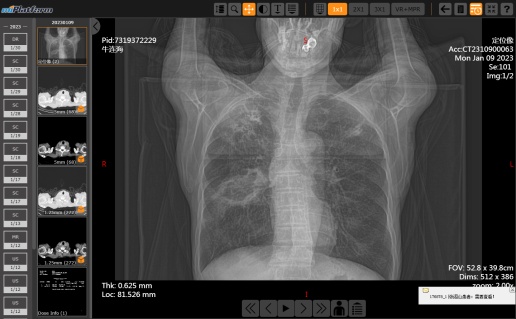 | 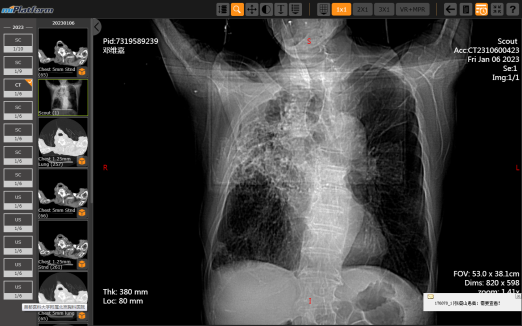 | 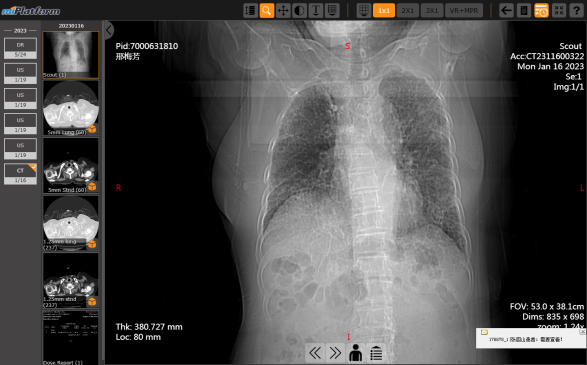 | | 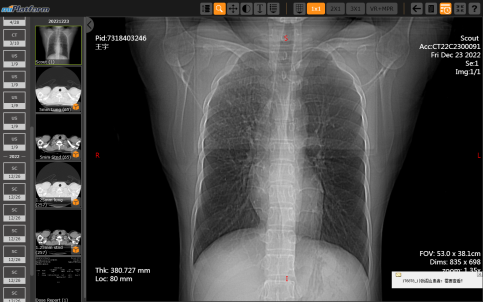 |
| Code | M1 | M2 | M3 | M4 | M5 | M6 | M7 | | M8 |
| Gender | Female | Male | Male | Male | Male | Male | Female | | Male |
| Age (years) | 65 | 55 | 47 | 35 | 67 | 75 | 87 | | 22 |
| Comorbidities | Rheumatoid arthritis,  Interstitial pneumonia | Hypertension,  Diabetes mellitus | Hypertension,  Diabetes mellitus,  Anemia | None | Renal insufficiency,  Anemia | Arrhythmia,  Anemia | Rheumatism, Interstitial pneumonia | | None |
| Laboratory markers | | | | | | | | | |
| WBC, 10^9^/L | 6.24 | 14.74 | 4.75 | 6.32 | 6.28 | 8.99 | 11.65 | | 7.6 |
| Hemoglobin, g/L | 113 | 105 | 77 | 77 | 78 | 100 | 111 | | 161 |
| Platelets, 10^9^/L | 312 | 331 | 229 | 417 | 316 | 283 | 169 | | 289 |
| Lymphocyte, % | 0.87 | 3.82 | 0.6 | 1.12 | 1.21 | 0.46 | 2.14 | | 2.16 |
| Hypersensitive C-reactive protein, mg/L | 49.42 | 95.51 | 53.37 | 73.94 | 18.41 | 123.27 | 108.02 | | 1.91 |
| D‐dimer, mg/L | 0.8 | 0.99 | 0.69 | 0.47 | 2.04 | 2.54 | 1.69 | | 0.11 |
| Albumin, g/dl | 32.9 | 30.2 | 35.9 | 33.8 | 25.2 | 32 | 26.9 | | 47.1 |
| Procalcitonin, ng/ml | 0.13 | 0.03 | 0.13 | 0.24 | 0.08 | 0.11 | 0.05 | | 0.02 |
| Creatinine, μmol/L | 34.2 | 69.9 | 95.1 | 55.9 | 3.54 | 44.1 | 39 | | 72.7 |
| LDH, U/L | 288 | 188 | 204 | 122 | 150 | 216 | 334 | | 182 |
| **TB laboratory confirmation** | | | | | | | | | |
| Sputum smear | Negative | Negative | Negative | Positive | Negative | Positive | Negative | | Negative |
| Liquid culture | Negative | Positive | Positive | Positive | Negative | Positive | Positive | | Positive |
| Gene Xpert | Positive | Positive | Positive | Positive | Positive | Positive | Positive | | Positive |
| **Severity of TB** | Non-severe | Non-severe | Extensive/Advanced | Extensive/Advanced | Extensive/Advanced | Extensive/Advanced | Non-severe | | Non-severe |
| Clinical outcome | Improvement | Improvement | Improvement | Improvement | Improvement | Improvement | Improvement | | Improvement |
|  |  |  |  |  |  |  |  | |  |
| Severe | | | | | | | | | |
| Chest X-ray or CT | 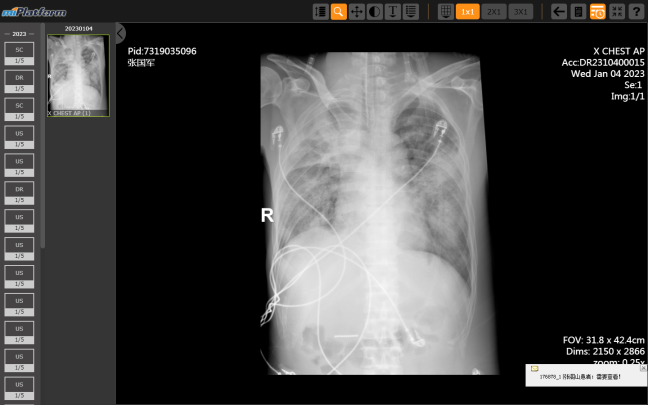 | 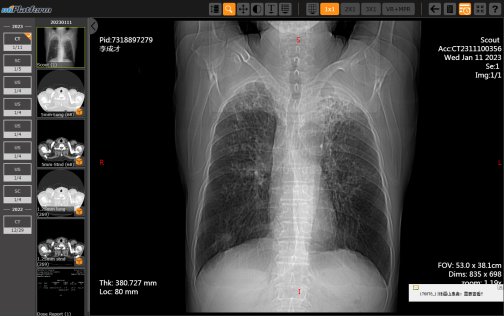 | 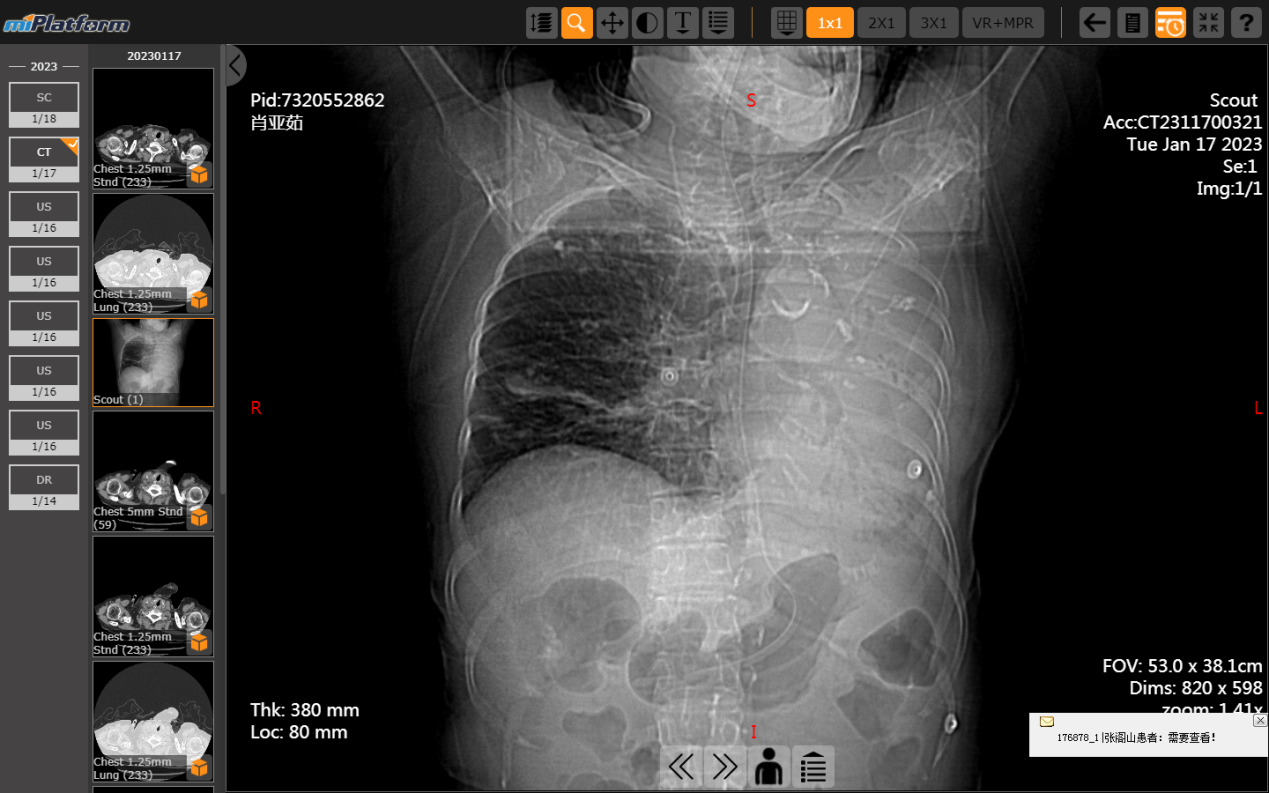 |  |  |  |  |  | |
| Code | S1 | S2 | S3 |  |  |  |  |  | |
| Gender | Male | Male | Female |  |  |  |  |  | |
| Age (years) | 55 | 73 | 77 |  |  |  |  |  | |
| Comorbidities | Diabetes mellitus,  Anemia,  Nephrotic syndrome | Diabetes mellitus,  Anemia | Coronary artery disease,  Diabetes mellitus,  Anemia |  |  |  |  |  | |
| Laboratory markers | | | | | | | | | |
| WBC, 10^9^/L | 19.4 | 7.72 | 7.57 |  |  |  |  |  | |
| Hemoglobin, g/L | 105 | 92 | 100 |  |  |  |  |  | |
| Platelets, 10^9^/L | 422 | 309 | 431 |  |  |  |  |  | |
| Lymphocyte, % | 0.95 | 0.67 | 0.79 |  |  |  |  |  | |
| Hypersensitive C-reactive protein, mg/L | 127.17 | 81.8 | 26.07 |  |  |  |  |  | |
| D‐dimer, mg/L | 22.66 | 4.35 | 1.38 |  |  |  |  |  | |
| Albumin, g/dl | 20 | 29.1 | 29.7 |  |  |  |  |  | |
| Procalcitonin, ng/ml | 0.44 | 0.53 | 0.03 |  |  |  |  |  | |
| Creatinine, μmol/L | 53.1 | 58.1 | 40.9 |  |  |  |  |  | |
| LDH, U/L | 217 | 166 | 172 |  |  |  |  |  | |
| **TB laboratory confirmation** | | | | | | | | | |
| Sputum smear | Negative | Positive | Negative |  |  |  |  |  | |
| Liquid culture | Negative | Positive | Positive |  |  |  |  |  | |
| Gene Xpert | Positive | Positive | Positive |  |  |  |  |  | |
| **Severity of TB** | Extensive/Advanced | Non-severe | Extensive/Advanced |  |  |  |  |  | |
| Clinical outcome | Dead | Improvement | ICU |  |  |  |  |  | |
